# WHO European Childhood Obesity Surveillance Initiative: body mass index and level of overweight among 6–9-year-old children from school year 2007/2008 to school year 2009/2010

**DOI:** 10.1186/1471-2458-14-806

**Published:** 2014-08-07

**Authors:** Trudy MA Wijnhoven, Joop MA van Raaij, Angela Spinelli, Gregor Starc, Maria Hassapidou, Igor Spiroski, Harry Rutter, Éva Martos, Ana I Rito, Ragnhild Hovengen, Napoleón Pérez-Farinós, Ausra Petrauskiene, Nazih Eldin, Lien Braeckevelt, Iveta Pudule, Marie Kunešová, João Breda

**Affiliations:** Division of Noncommunicable Diseases and Life-course, World Health Organization Regional Office for Europe, UN City, Marmorvej 51, DK-2100 Copenhagen Ø, Denmark; Centre for Nutrition, Prevention and Health Services, National Institute for Public Health and the Environment, PO Box 1, 3720 BA Bilthoven, The Netherlands; Division of Human Nutrition, Wageningen University, PO Box 8129, 6700 EV Wageningen, The Netherlands; National Centre for Epidemiology, Surveillance and Health Promotion, National Institute of Health, Viale Regina Elena 299, I-00161 Rome, Italy; Faculty of Sport, University of Ljubljana, Gortanova 22, 1000 Ljubljana, Slovenia; Department of Nutrition and Dietetics, Alexander Technological Educational Institute of Thessaloniki, PO Box 14561, 54101 Thessaloniki, Greece; Department of Physiology and Monitoring of Nutrition, Institute for Public Health of the Republic of Macedonia, 50 Divizija 6, 1000 Skopje, the former Yugoslav Republic of Macedonia; Faculty of Public Health and Policy, London School of Hygiene and Tropical Medicine, 15-17 Tavistock Place, London, WC1H 9SH United Kingdom; National Institute for Food and Nutrition Science, Gyali ut 3/a., 1097 Budapest, Hungary; National Institute of Health Doutor Ricardo Jorge IP, Av. Padre Cruz, 1649-016 Lisbon, Portugal; Department of Health Statistics, Norwegian Institute of Public Health, PO Box 4404, Nydalen, N-0403 Oslo, Norway; Spanish Agency for Consumer Affairs, Food Safety and Nutrition, Alcala 56, 28071 Madrid, Spain; Department of Preventive Medicine, Lithuanian University of Health Sciences, Eiveniu str. 4, 50009 Kaunas, Lithuania; Health Promotion Department, Health Service Executive, Railway St, Navan, County Meath, Ireland; National Nutrition Surveillance Centre, School of Public Health, Physiotherapy & Population Science, University College Dublin, Belfield, Dublin, 4, Ireland; Flemish Agency for Care and Health, Flemish Ministry of Welfare, Public Health and Family, Koning Albert II-Laan 35, PO Box 33, 1030 Brussels, Belgium; Centre for Disease Prevention and Control, 22 Duntes Street, LV-1005 Riga, Latvia; Obesity Management Centre, Institute of Endocrinology, Narodni 8, 116 94 Prague 1, Czech Republic

## Abstract

**Background:**

The World Health Organization (WHO) Regional Office for Europe has established the Childhood Obesity Surveillance Initiative (COSI) to monitor changes in overweight in primary-school children. The aims of this paper are to present the anthropometric results of COSI Round 2 (2009/2010) and to explore changes in body mass index (BMI) and overweight among children within and across nine countries from school years 2007/2008 to 2009/2010.

**Methods:**

Using cross-sectional nationally representative samples of 6−9-year-olds, BMI, anthropometric Z-scores and overweight prevalence were derived from measured weight and height. Significant changes between rounds were assessed using variance and t-tests analyses.

**Results:**

At Round 2, the prevalence of overweight (including obesity; WHO definitions) ranged from 18% to 57% among boys and from 18% to 50% among girls; 6 − 31% of boys and 5 − 21% of girls were obese. Southern European countries had the highest overweight prevalence. Between rounds, the absolute change in mean BMI (range: from −0.4 to +0.3) and BMI-for-age Z-scores (range: from −0.21 to +0.14) varied statistically significantly across countries. The highest significant decrease in BMI-for-age Z-scores was found in countries with higher absolute BMI values and the highest significant increase in countries with lower BMI values. The highest significant decrease in overweight prevalence was observed in Italy, Portugal and Slovenia and the highest significant increase in Latvia and Norway.

**Conclusions:**

Changes in BMI and prevalence of overweight over a two-year period varied significantly among European countries. It may be that countries with higher prevalence of overweight in COSI Round 1 have implemented interventions to try to remedy this situation.

**Electronic supplementary material:**

The online version of this article (doi:10.1186/1471-2458-14-806) contains supplementary material, which is available to authorized users.

## Background

Through the European Charter on Counteracting Obesity, Member States in the European Region of the World Health Organization (WHO) declared in 2006 their commitment to strengthen action on counteracting obesity and to place this issue high on the political agenda of their governments. Article 2.2 of the Charter states that “Curbing the epidemic and reversing the trend are the ultimate goal of action in the Region. Visible progress, especially relating to children and adolescents, should be achievable in most countries in the next 4–5 years and it should be possible to reverse the trend by 2015 at the latest”. Moreover, article 3.2 specifies that “A process needs to be put together to develop internationally comparable core indicators for inclusion in national health surveillance systems. These data could then be used for advocacy, policy-making and monitoring purposes. This would also allow for regular evaluation and review of policies and actions and for the dissemination of findings to a wide audience” [1]. The establishment of the WHO European Childhood Obesity Surveillance Initiative (COSI) in 2006 was a response to this Charter, which was signed at the WHO European Ministerial Conference on Counteracting Obesity in Istanbul, Turkey [2]. The importance of such surveillance mechanisms was reinforced as one of the strongest dimensions in the Vienna Declaration on Nutrition and Noncommunicable Diseases in the Context of Health 2020 [3], which was endorsed at the sixty-third session of the WHO Regional Committee for Europe in September 2013 [4].

COSI routinely measures overweight and obesity prevalence of primary-school children aged 6–9 years, in order to monitor progress with curbing excess body weight in this population group and to permit inter-country comparisons within the WHO European Region. The first COSI data collection round took place during the school year 2007/2008 (Round 1) in which thirteen countries participated [5]. A second COSI data collection round took place during the school year 2009/2010 (Round 2) and some of the participating countries have already published the national data analyses of Round 2 [6–10].

The aim of this paper is twofold: 1) to present the findings of COSI Round 2 and investigate whether differences exist in mean values of anthropometric measurements (weight, height), indices (body mass index (BMI) and Z-scores) and prevalence estimates across countries and between boys and girls; and 2) to assess which countries are on track to achieving the Charter’s ultimate goal by studying the direction and magnitude of the change in mean anthropometric values and overweight prevalence estimates from Round 1 to Round 2.

## Methods

COSI Round 2 (2009/2010) was conducted in fifteen countries: Belgium, Cyprus, Czech Republic, Greece, Hungary, Ireland, Italy, Latvia, Lithuania, Malta, Norway, Portugal, Slovenia, Spain and the former Yugoslav Republic of Macedonia. All Round 2 countries except for Cyprus and Malta delivered their 2009/2010 data to the WHO-COSI database in line with the COSI protocol and thus data from 13 countries were used for the present analysis. The implementation characteristics of COSI Round 1 (2007/2008) were described in detail elsewhere [5].

### Protocol development

A common protocol was developed throughout 2007 by the WHO Regional Office for Europe and Member States participating in COSI, and it was used for both rounds [11]. Two protocol changes in recording procedures were made for Round 2: precise recording of the time of measurement was made optional, and a mandatory variable to indicate whether measurements took place before or after lunch was added. In addition, children were no longer routinely asked to go to the toilet before the measurements [12].

### Study population and sampling design

Countries that participated in Round 1 could decide for Round 2 to select a new nationally representative sample of schools or to go back to the same schools that were selected in Round 1 and select randomly the classes at these sentinel sites. Four countries (Ireland, Lithuania, Norway and Portugal) chose the sentinel site approach. The entire population of interest was included by Belgium (Flanders only) and new nationally representative samples were taken by the other eight countries (see Additional file 1).

#### Age groups chosen

Given the differences among countries in school systems, the age of children entering the first class of primary school (reception year), and the number of children repeating a grade, it was difficult to implement a uniform sampling approach that was applicable in every country. Age was therefore taken as the first condition for the sampling procedures. Countries were free to select one or more of the following four defined COSI age groups: 6.0–6.9, 7.0–7.9, 8.0–8.9 or 9.0–9.9 years. Since children of this age in all countries are enrolled in primary schools, the school population was therefore taken to be representative of the total population in these age groups.

#### Stratification

Stratification of the primary sampling unit (PSU) was applied if it was expected that differences in anthropometric measurements and indices across strata would be observed. This was done by eight countries: the Czech Republic by region and level of urbanization; Greece by prefecture; Hungary by county; Italy by region; Latvia by level of urbanization and language of instruction; Lithuania by district and level of urbanization; Spain by geopolitical region and size of city/village; and the former Yugoslav Republic of Macedonia by level of urbanization.

#### Sampling units

Cluster sampling was employed by the eight countries that drew a new sample whereby the PSU was the primary school or the class (except in the Czech Republic, where the PSU was composed of paediatric clinics, since COSI was attached to the mandatory health checks that are performed by paediatricians). Primary schools and classes were selected randomly from the list of all primary schools (public, private and special schools) centrally available in each country through the Ministry of Education or at the national school registry (or national list of primary care paediatricians). If less than about 1% of the target children were enrolled in private or special schools (e.g. schools for children with learning disability or visual impairment), countries had the choice of excluding these schools from the sampling frame. If the majority of the children of the targeted age group were in the same grade, then the class was drawn from within that grade level. If the targeted age group was spread across grades, however, all grades where most children from this age group were present were sampled. In every sampled class, all children were invited to participate.

#### Sample size

Rudolf *et al*. suggest using the standard deviation (SD) scores or Z-scores of a BMI distribution to demonstrate whether a halt in the rise in overweight or obesity is achieved [13]. The calculated sample size of ≈ 2300 children per age group was based on an 80% power to detect a minimum difference of 0.10 Z-score in mean BMI per year at a two-sided 5% significance level. To achieve the same precision with a cluster sample design as with a simple random sample, the minimum final effective sample size should be ≈ 2800 children per age group, whereby a design effect of 1.2 was applied [14]. Additional file 2 shows by country the number of children that were invited to participate in Round 2; that were measured; that had complete information on age, sex, weight and height; and that fell within the targeted age group. A total of 224 920 children aged 6–9 years were included in the data analyses (114 457 boys and 110 463 girls).

### Data collection procedures

Countries decided on the measurement period. Data collection, however, was avoided during the first two weeks of a school term or immediately after a major holiday. Taking the local arrangements, circumstances and budget into account, countries chose the most appropriate professionals to collect data from the children, hereafter called examiners. Additional file 3 gives a summary of the application of the COSI protocol characteristics in each country in Round 2.

#### Ethics approval procedures

The COSI protocol is in accordance with the international Ethical Guidelines for Biomedical Research Involving Human Subjects [15]. Depending on local circumstances, the procedures were also approved by local ethical committees. This was not needed in four countries because data collection procedures were part of legislation (Belgium), a compulsory school programme (Slovenia), a National Annual Program of Public Health (the former Yugoslav Republic of Macedonia) or were regulated by the National Health Authority and Regional Health Authorities (Spain). The local committees in the other nine countries were: Czech Republic: the Institutional Ethical Committee of the Institute of Endocrinology; Greece: the Ethics Committee of the Alexander Technological Educational Institute of Thessaloniki; Hungary: the Scientific and Research Ethics Committee of the Medical Research Council; Ireland: the Research Ethics Committee of the University College Dublin; Italy: the Institutional Ethical Committee of the Italian National Institute of Health; Latvia: Central Medical Ethics Committee; Lithuania: Lithuanian Bioethics Committee; Norway: Regional Committee for Medical and Health Research Ethics; and Portugal: Portuguese Data Protection Authority. Detailed information on the ethical clearance procedures in each country is included in Additional file 4. Parents were always fully informed about all study procedures and their informed consent approach (passive or active by one or two parents) was obtained. Children’s consent was always obtained prior to the measurements.

#### Anthropometric measurements

Prior to data collection, all examiners were trained in measuring weight and height using WHO standardized techniques [16]. Children were asked to take off their shoes and socks as well as all heavy clothing (coats, sweaters, jackets, etc.) and to remove items such as wallets, mobile phones or key chains. Body weight was measured to the nearest 0.1 kg with portable digital (mainly manufacturer-calibrated) scales and body height was measured standing upright to the nearest 0.1 cm with portable stadiometers. Body weight was then adjusted for the weight of the clothes worn. The average weights of types of clothing were provided by each country. Where possible, the same anthropometric equipment was used throughout a country. Anthropometric measurements were preferably done in the mornings before lunch time, although this had not always been feasible.

### Data elaboration

All country datasets were reviewed in a standard manner at the Regional Office for inconsistencies and completeness before they were merged for the intercountry analyses. The final dataset included children with informed consent and complete information on age, sex, weight and height. Children were excluded from the final dataset if their age did not fall within the targeted age group.

The child’s age (in years) was calculated using the formula: (date of measurement minus date of birth (expressed in days))/365.25. When a complete date of birth was not provided but only the month and year of birth, then the child’s age (in years) was calculated by dividing the number of months between the date of birth and the date of measurement by 12 (this was done for the entire Belgian dataset). BMI was calculated using the formula: weight (kg) divided by height squared (m^2^).

The 2007 WHO recommended cut-offs for school-age children and adolescents were used to compute height-for-age (H/A), weight-for-age (W/A) and BMI-for-age (BMI/A) Z-scores, and to interpret anthropometric indicators [17, 18], whereby stunting and severe stunting were defined as the proportion of children with a H/A value below −2 Z-scores and below −3 Z-scores, respectively, relative to the 2007 WHO growth reference median [17]. Underweight and severe underweight were defined as the proportion of children with a W/A value below −2 Z-scores and below −3 Z-scores, respectively. Thinness and severe thinness were defined as the proportion of children with a BMI/A value below −2 Z-scores and below −3 Z-scores, respectively. Overweight and obesity were defined as the proportion of children with a BMI/A value above +1 Z-score and above +2 Z-scores, respectively. Overweight and obesity were also estimated using the IOTF cut-off points [19], as they are widely used in the WHO European Region (see Additional file 5).

According to WHO definitions, the prevalence estimates for stunted children include those who are severely stunted, the prevalence estimates for underweight children include those who are severely underweight, the prevalence estimates for thin children include those who are severely thin, and the prevalence estimates for overweight children include those who are obese [16].

Children with biologically implausible (or extreme) values were excluded from the analysis [18]: W/A values below −6 or above +5 Z-scores; H/A values below −6 or above +6 Z-scores; and BMI/A values below −5 or above +5 Z-scores relative to the 2007 WHO growth reference median.

### Statistical analysis

A *P*-value of 0.05 was used to define statistical significance. All statistical analyses, except the Games-Howell *post hoc* tests, were performed in Stata version 10.1 (StataCorp, College Station, TX, USA). The latter was performed in SPSS version 20.0 (IBM, Armonk, NY, USA).

#### Round 2

Sampling weights to adjust for the applied sampling design, oversampling and non-response rate of Round 2 were available for only four countries. For the other countries, these could not be calculated for various reasons. For instance, the registration of the children in schools and classes was not entirely complete. Hence, the analyses were performed unweighted. Means ± SDs were calculated for all measurements (weight and height) and anthropometric indices (BMI, W/A, H/A and BMI/A Z-scores) by age group, sex and country. For each country-specific dataset, these six continuous variables were tested by age group for normality using normal quantile–quantile plots. Weight and BMI were found to be highly positively skewed in all datasets. They were therefore transformed to attain normality and their transformed values were used for the intercountry comparisons. Using the command ‘ladder’ in Stata, the best option suggested was inverse transformation for weight and 1/square transformation for BMI for the majority of the datasets. Although the distribution of W/A and BMI/A Z-scores was also skewed to the right, the command ‘ladder’ showed no need to apply transformations to normalize them.

The homogeneity of variances was tested using Levene’s test [20]. Since the data showed heterogeneity of variances between countries and because of an unbalanced design (unequal group sizes), the main effects of country and sex and their interaction on all mean anthropometric values was assessed using two-way analysis of variance (ANOVA), with the Games-Howell *post hoc* test for the multiple comparisons between countries [21]. This was done separately for all age groups because not every country had included all age groups. In the case of an interaction effect, a one-way ANOVA was performed to assess significant differences across countries by sex and between sexes by country for all four age groups.

Prevalence estimates are presented as percentages. Within each age group, the chi-squared test was used to determine differences in the prevalence estimates across countries for the total group and for boys and girls separately. If the chi-squared test was found significant, the Marascuilo procedure was used for the multi-group comparisons of proportions between countries [22]. In addition a chi-squared test was used to determine the statistical significance of differences in the prevalence estimates between boys and girls within each age group as well as to assess a linear trend in the prevalence estimates with increasing age for the seven countries with multiple age groups selected (Belgium, Greece, Ireland, Italy, Lithuania, Slovenia and Spain).

#### Changes from Round 1 to Round 2

The absolute change in mean values for all measurements (weight and height) and anthropometric indices (BMI, W/A, H/A and BMI/A Z-scores) was calculated for the nine countries that participated in both rounds (Belgium, Czech Republic, Ireland, Italy, Latvia, Lithuania, Norway, Portugal and Slovenia), by age group and sex. A two-way ANOVA was applied to assess the interaction effect of country and round on all mean anthropometric values, for boys and girls separately. In the case of no significant interaction effect, two-way ANOVA without the interaction term (additive model) was performed to assess the main effects of country and round on the values. By age group and for boys and girls separately, Levene’s test [20] was applied to assess the homogeneity of variances between the two rounds. If a significant interaction effect was found by the two-way ANOVA, the unpaired t-test (equal variance) or the unpaired Welch’s t-test (unequal variance) was performed to assess whether the difference in mean values between the two rounds in each country was statistically significant.

A *z* test for two independent proportions was used to determine the statistical significance of differences in the prevalence estimates between the two rounds by country and age group.

## Results

### COSI Round 2 (2009/2010)

#### Weight, height, BMI, W/A, H/A and BMI/A Z-scores

Mean values for weight and W/A Z-score are presented in Table 1, mean values for height and H/A Z-score in Table 2 and mean values for BMI and BMI/A Z-score in Table 3. All mean Z-scores were positive. Weight and height increased with age and boys were taller and heavier than girls in all age groups (where statistically significant differences between boys and girls were found). Based on the values found in countries that targeted multiple age groups, BMI also increased with age (except in Italian girls). Mean BMI/A Z-scores close to the +1 Z-score curve values of the 2007 WHO growth reference were found in three countries (Greece, Italy and Spain). Median values of weight and BMI are available in Additional file 6.

**Table 1:** **Weight and weight-for-age Z-scores** ^*^ **of boys and girls aged 6–9 years in COSI Round 2 (2009/2010), by age and country** ^**¶**^

Age group and country ^‡^	Weight ^#^ (kg)		W/A Z-score	
	Boys	Girls	Boys	Girls
	Mean (SD)			
6-year-olds	†	†	†	†
BEL	23.0 (3.7)^§a^	22.7 (3.9)^a^	0.35 (1.06)^a^	0.33 (1.00)^a^
SVN	24.9 (4.6)^§b^	24.4 (4.6)^b^	0.70 (1.20)^§b^	0.62 (1.10)^b^
ESP	24.9 (4.6)^§b^	24.5 (4.6)^b^	0.81 (1.19)^b^	0.72 (1.09)^c^
7-year-olds	†	†	†	†
BEL	25.8 (5.0)^§a^	25.7 (5.4)^a^	0.40 (1.20)^a^	0.40 (1.13)^a^
CZE	26.0 (4.8)^§ab^	25.3 (5.1)^a^	0.63 (1.17)^§bc^	0.51 (1.12)^abc^
GRC	29.5 (5.8)^§c^	29.1 (6.1)^b^	1.17 (1.27)^§d^	1.05 (1.15)^d^
HUN	26.7 (5.5)^bde^	26.4 (5.8)^ac^	0.57 (1.28)^ab^	0.54 (1.22)^abc^
IRL	26.0 (4.2)^§ad^	25.6 (4.6)^ac^	0.53 (1.04)^ab^	0.51 (1.02)^abc^
LVA	27.4 (5.3)^§fg^	26.5 (5.3)^cd^	0.63 (1.18)^§b^	0.45 (1.08)^ac^
LTU	27.6 (5.3)^§f^	27.0 (5.5)^d^	0.68 (1.17)^§bc^	0.55 (1.09)^bc^
PRT	27.0 (5.5)^deg^	27.2 (5.7)^de^	0.61 (1.25)^b^	0.66 (1.15)^be^
SVN	27.8 (5.7)^§f^	27.1 (5.8)^d^	0.78 (1.25)^§c^	0.64 (1.14)^b^
ESP	28.5 (5.7)^§h^	27.9 (5.8)^e^	0.96 (1.24)^§e^	0.81 (1.13)^e^
MKD	27.6 (6.4)^§ef^	26.5 (6.4)^c^	0.70 (1.46)^§bc^	0.50 (1.32)^ac^
8-year-olds	†	†	†	†
BEL	29.3 (5.5)^§a^	29.1 (5.9)^a^	0.45 (1.10)^§a^	0.40 (1.06)^a^
ITA	32.3 (7.3)^§b^	31.6 (7.3)^b^	0.90 (1.31)^§b^	0.70 (1.22)^b^
NOR	30.2 (5.7)^§c^	29.6 (5.6)^c^	0.69 (1.11)^§c^	0.56 (1.00)^c^
SVN	31.8 (7.0)^§d^	31.1 (7.1)^b^	0.90 (1.26)^§b^	0.72 (1.16)^b^
ESP	31.7 (6.4)^bd^	31.5 (6.7)^b^	0.91 (1.18)^§b^	0.79 (1.12)^b^
9-year-olds	†	†	†	†
BEL	32.8 (7.3)^a^	32.9 (7.6)^a^	0.46 (1.18)^§a^	0.36 (1.18)^a^
GRC	38.4 (8.9)^§b^	37.6 (9.1)^b^	1.30 (1.21)^§b^	1.02 (1.23)^b^
IRL	33.0 (6.3)^§ac^	32.2 (6.9)^a^	0.63 (1.05)^§c^	0.37 (1.13)^ac^
ITA	33.9 (7.8)^§c^	32.9 (7.6)^a^	0.79 (1.28)^§d^	0.53 (1.21)^c^
LTU	34.7 (7.5)^§d^	33.7 (7.3)^c^	0.66 (1.12)^§c^	0.40 (1.09)^a^
SVN	34.2 (7.9)^cd^	33.8 (8.0)^c^	0.85 (1.24)^§de^	0.68 (1.23)^d^
ESP	35.9 (7.7)^§e^	35.2 (7.7)^d^	0.97 (1.13)^§e^	0.73 (1.12)^d^

*Abbreviations:* *ANOVA* analysis of variance, *COSI* Childhood Obesity Surveillance Initiative, *SD* standard deviation, *W/A* weight-for-age, *WHO* World Health Organization.

^*^Based on the 2007 WHO growth reference for school-age children and adolescents [17].

^¶^Body weight was adjusted for clothes worn when measured and children with a W/A Z-score < −6 or > +5 were excluded.

^‡^The country codes refer to the International Organization for Standardization (ISO) 3166–1 Alpha-3 country codes and countries were listed in alphabetical order by their full names: BEL, Belgium (Flanders); CZE, Czech Republic; GRC, Greece; HUN, Hungary; IRL, Ireland; ITA, Italy; LVA, Latvia; LTU, Lithuania; NOR, Norway; PRT, Portugal (all regions except Madeira); SVN, Slovenia; ESP, Spain; MKD, the former Yugoslav Republic of Macedonia.

^#^Non-normally distributed and underwent inverse transformation prior to ANOVA and Games-Howell *post hoc* tests.

†Statistically significant difference of mean value across countries for the indicated age group (one-way ANOVA; P < 0.0001).

^§^Statistically significant difference of mean value between boys and girls for the indicated country (one-way ANOVA; P ≤ 0.05).

^a,b,c,d,e,f,g,h^Within each sex-age group (e.g. 6-year-old girls), mean values that share the same superscript letter do not statistically significantly differ from each other (Games-Howell *post hoc* test). For example, for the 6-year-old girls, each mean W/A Z-score value is significantly different from the other two whereas the value of Belgian 6-year-old boys differed significantly from the other two and no significant difference was found between Slovenian and Spanish 6-year-old boys.

**Table 2:** **Height and height-for-age Z-scores** ^*^ **of boys and girls aged 6–9 years in COSI Round 2 (2009/2010), by age and country** ^**¶**^

Age group and country ^‡^	Height (cm)		H/A Z-score	
	Boys	Girls	Boys	Girls
	Mean (SD)			
6-year-olds	†	†	†	†
BEL	120.5 (5.3)^§a^	119.6 (5.3)^a^	0.36 (0.99)^§a^	0.34 (0.95)^a^
SVN	124.4 (5.3)^§b^	123.5 (5.5)^b^	0.87 (0.99)^b^	0.88 (0.99)^b^
ESP	121.4 (5.5)^§c^	120.5 (5.5)^c^	0.45 (1.01)^c^	0.41 (1.00)^a^
7-year-olds	†	†	†	†
BEL	125.6 (6.0)^§a^	124.7 (6.0)^a^	0.32 (1.02)^§a^	0.29 (1.00)^a^
CZE	126.0 (5.4)^§abc^	124.8 (5.7)^abc^	0.65 (1.00)^bcde^	0.57 (1.04)^bc^
GRC	128.5 (5.8)^§d^	127.8 (5.9)^de^	0.71 (1.07)^bf^	0.70 (1.03)^cd^
HUN	127.2 (5.9)^§ef^	126.0 (5.9)^f^	0.56 (1.07)^beg^	0.51 (1.04)^be^
IRL	125.5 (5.2)^§ac^	123.8 (5.4)^b^	0.35 (0.93)^ah^	0.23 (0.96)^af^
LVA	128.7 (5.6)^§d^	127.3 (5.7)^d^	0.62 (1.00)^§be^	0.51 (0.98)^be^
LTU	129.5 (5.7)^§g^	128.6 (5.8)^g^	0.76 (1.02)^cf^	0.72 (1.00)^d^
PRT	126.4 (5.5)^§bce^	125.5 (5.7)^cf^	0.35 (0.97)^ah^	0.33 (0.98)^af^
SVN	129.0 (5.5)^§dg^	128.1 (5.6)^eg^	0.81 (0.95)^f^	0.78 (0.95)^d^
ESP	127.1 (5.6)^§ef^	126.0 (5.7)^f^	0.47 (0.98)^gh^	0.40 (0.96)^ef^
MKD	127.3 (6.4)^§f^	126.2 (6.4)^f^	0.52 (1.16)^deg^	0.48 (1.11)^be^
8-year-olds	†	†	†	†
BEL	132.6 (5.9)^§a^	131.8 (6.0)^a^	0.48 (0.98)^§a^	0.41 (0.98)^ab^
ITA	133.1 (6.0)^§b^	132.0 (6.1)^b^	0.41 (1.00)^§b^	0.29 (1.01)^c^
NOR	133.1 (5.7)^§bc^	131.8 (5.8)^ab^	0.63 (0.96)^§c^	0.48 (0.94)^a^
SVN	134.4 (5.8)^§d^	133.6 (5.8)^c^	0.76 (0.95)^§d^	0.68 (0.94)^d^
ESP	132.4 (6.0)^§ac^	131.8 (5.9)^ab^	0.42 (0.99)^ab^	0.37 (0.96)^bc^
9-year-olds	†	†	†	†
BEL	137.3 (6.5)^§a^	136.9 (6.8)^a^	0.40 (1.01)^§a^	0.28 (1.03)^a^
GRC	139.9 (6.7)^§b^	139.3 (6.7)^b^	0.74 (1.07)^§b^	0.59 (1.07)^b^
IRL	136.5 (5.8)^§c^	134.9 (6.5)^c^	0.37 (0.94)^§ac^	0.11 (1.04)^c^
ITA	135.6 (6.0)^§c^	134.7 (6.3)^c^	0.32 (0.99)^§c^	0.15 (1.02)^c^
LTU	140.5 (6.3)^§b^	139.9 (6.4)^b^	0.72 (1.00)^§b^	0.57 (0.99)^b^
SVN	137.7 (6.0)^§a^	137.0 (6.2)^a^	0.67 (0.99)^§b^	0.55 (1.02)^b^
ESP	137.8 (6.2)^§a^	136.9 (6.6)^a^	0.43 (0.96)^§a^	0.25 (0.99)^ac^

*Abbreviations:* *ANOVA* analysis of variance, *COSI* Childhood Obesity Surveillance Initiative, *H/A* height-for-age, *SD* standard deviation, *WHO* World Health Organization.

^*^Based on the 2007 WHO growth reference for school-age children and adolescents [17].

^¶^Children with a H/A Z-score < −6 or > +6 were excluded.

^‡^The country codes refer to the International Organization for Standardization (ISO) 3166–1 Alpha-3 country codes and countries were listed in alphabetical order by their full names: BEL, Belgium (Flanders); CZE, Czech Republic; GRC, Greece; HUN, Hungary; IRL, Ireland; ITA, Italy; LVA, Latvia; LTU, Lithuania; NOR, Norway; PRT, Portugal (all regions except Madeira); SVN, Slovenia; ESP, Spain; MKD, the former Yugoslav Republic of Macedonia.

†Statistically significant difference of mean value across countries for the indicated age group (one-way ANOVA; P < 0.0001).

^§^Statistically significant difference of mean value between boys and girls for the indicated country (one-way ANOVA; P < 0.05).

^a,b,c,d,e,f,g,h^Within each sex-age group (e.g. 6-year-old boys), mean values that share the same superscript letter do not statistically significantly differ from each other (Games-Howell *post hoc* test). For example, for the 6-year-old boys, each mean H/A Z-score value is significantly different from the other two whereas the value of Slovenian 6-year-old girls differed significantly from the other two and no significant difference was found between Belgian and Spanish 6-year-old girls.

**Table 3:** **BMI and BMI-for-age Z-scores** ^*^ **of boys and girls aged 6–9 years in COSI Round 2 (2009/2010), by age and country** ^**¶**^

Age group and country ^‡^	BMI ^#^ (kg/m ^2^ )		BMI/A Z-score	
	Boys	Girls	Boys	Girls
	Mean (SD)			
6-year-olds	†	†	†	†
BEL	15.8 (1.7)^a^	15.8 (1.9)^a^	0.16 (1.08)^a^	0.16 (1.02)^a^
SVN	16.0 (2.2)^a^	15.9 (2.2)^a^	0.21 (1.34)^a^	0.15 (1.16)^a^
ESP	16.8 (2.2)^b^	16.8 (2.4)^b^	0.78 (1.25)^§b^	0.66 (1.15)^b^
7-year-olds	†	†	†	†
BEL	16.3 (2.2)^a^	16.4 (2.5)^a^	0.27 (1.23)^a^	0.29 (1.14)^a^
CZE	16.3 (2.3)^ab^	16.2 (2.4)^a^	0.31 (1.29)^ab^	0.23 (1.16)^ab^
GRC	17.7 (2.7)^c^	17.7 (2.8)^b^	1.06 (1.31)^§c^	0.90 (1.15)^c^
HUN	16.4 (2.5)^abde^	16.5 (2.7)^ac^	0.30 (1.37)^ab^	0.33 (1.23)^abd^
IRL	16.5 (2.0)^def^	16.6 (2.1)^cd^	0.46 (1.05)^bd^	0.50 (1.00)^de^
LVA	16.5 (2.3)^§abeg^	16.2 (2.4)^a^	0.34 (1.25)^§ab^	0.18 (1.12)^b^
LTU	16.4 (2.4)^§abe^	16.2 (2.5)^a^	0.31 (1.27)^§ab^	0.17 (1.15)^b^
PRT	16.8 (2.5)^§f^	17.1 (2.7)^de^	0.56 (1.28)^d^	0.64 (1.15)^ef^
SVN	16.6 (2.6)^§beg^	16.4 (2.6)^a^	0.40 (1.38)^§bd^	0.25 (1.22)^ab^
ESP	17.5 (2.7)^c^	17.4 (2.7)^be^	0.94 (1.31)^§c^	0.76 (1.18)^cf^
MKD	16.9 (2.9)^§fg^	16.5 (2.9)^a^	0.55 (1.54)^§d^	0.29 (1.33)^ab^
8-year-olds	†	†	†	†
BEL	16.5 (2.3)^§a^	16.7 (2.5)^a^	0.21 (1.17)^a^	0.22 (1.09)^a^
ITA	18.1 (3.1)^§b^	18.0 (3.2)^b^	0.89 (1.37)^§b^	0.70 (1.23)^b^
NOR	16.9 (2.5)^c^	16.9 (2.3)^c^	0.43 (1.23)^c^	0.39 (1.01)^c^
SVN	17.5 (3.0)^§d^	17.3 (3.0)^c^	0.64 (1.37)^§d^	0.46 (1.22)^c^
ESP	18.0 (2.7)^b^	18.0 (2.9)^b^	0.92 (1.23)^§b^	0.77 (1.11)^b^
9-year-olds	†	†	†	†
BEL	17.3 (2.9)^a^	17.4 (3.0)^a^	0.31 (1.28)^§a^	0.26 (1.19)^a^
GRC	19.5 (3.5)^§b^	19.2 (3.6)^b^	1.26 (1.26)^§b^	0.93 (1.23)^b^
IRL	17.6 (2.5)^c^	17.5 (2.8)^ac^	0.58 (1.09)^§c^	0.41 (1.09)^ac^
ITA	18.3 (3.3)^§d^	18.0 (3.2)^c^	0.83 (1.38)^§d^	0.57 (1.22)^d^
LTU	17.5 (2.9)^§c^	17.1 (2.8)^d^	0.37 (1.23)^§a^	0.09 (1.16)^e^
SVN	17.8 (3.1)^c^	17.9 (3.4)^c^	0.64 (1.32)^§c^	0.51 (1.25)^cd^
ESP	18.8 (3.1)^e^	18.6 (3.2)^e^	1.00 (1.21)^§e^	0.76 (1.13)^f^

*Abbreviations:* *ANOVA* analysis of variance, *BMI* body mass index, *BMI/A* BMI-for-age, *COSI* Childhood Obesity Surveillance Initiative, *SD* standard deviation, *WHO* World Health Organization.

^*^Based on the 2007 WHO growth reference for school-age children and adolescents [17].

^¶^Body weight was adjusted for clothes worn when measured and children with a BMI/A Z-score < −5 or > +5 were excluded.

^‡^The country codes refer to the International Organization for Standardization (ISO) 3166–1 Alpha-3 country codes and countries were listed in alphabetical order by their full names: BEL, Belgium (Flanders); CZE, Czech Republic; GRC, Greece; HUN, Hungary; IRL, Ireland; ITA, Italy; LVA, Latvia; LTU, Lithuania; NOR, Norway; PRT, Portugal (all regions except Madeira); SVN, Slovenia; ESP, Spain; MKD, the former Yugoslav Republic of Macedonia.

^#^Non-normally distributed and underwent 1/square transformation prior to ANOVA and Games-Howell *post hoc* tests.

†Statistically significant difference of mean value across countries for the indicated age group (one-way ANOVA; P < 0.0001).

^§^Statistically significant difference of mean value between boys and girls for the indicated country (one-way ANOVA; P < 0.05).

^a,b,c,d,e^Within each sex-age group (e.g. 9-year-old boys), mean values that share the same letter superscript do not statistically significantly differ from each other (Games-Howell *post hoc* test). For example, for the 9-year-old boys, the mean BMI/A Z-scores of Greek, Italian and Spanish boys significantly differ from the other six, whereas the values of Belgian and Lithuanian boys do not differ significantly from each other as well as the values of Irish and Slovenian boys.

Two-way ANOVA showed a statistically significant interaction effect of country and sex on most anthropometric values (BMI/A Z-score for each of the four age groups (*P* < 0.05); inverse-transformed weight (*P* < 0.001), W/A Z-score (*P* < 0.0001) and 1/square-transformed BMI (*P* < 0.0001) for the 7-, 8- and 9-year-old groups; H/A Z-score for the 8-year-old group (*P* < 0.05) and height for the 8- and 9-year-old groups (*P* < 0.05)). Because significant results were found in all age groups, one-way ANOVA analyses were performed to assess within each age group the country effect for boys and girls separately and the sex effect for each country separately. Results from the one-way ANOVA analyses are also presented in Tables 1, 2 and 3. The main effect of country (*P* < 0.0001) on all mean values and the main effect of sex (*P* < 0.05) on most mean values were statistically significant. Subsequently, Games-Howell *post hoc* tests were performed for boys and girls separately within each age group to study the differences between countries in more detail (countries within each sex-age group that share the same superscript letter do not statistically significantly differ from each other).

Based on mean BMI/A Z-score values, three categories of countries could be determined. One group of countries (Belgium, Czech Republic, Hungary, Latvia, Lithuania and Norway) consistently had values (in both boys and girls as well as in multiple age groups when applicable) between the expected 2007 WHO growth reference value and 0.5 SD away from this reference median value. A second group of countries (Greece, Italy, Portugal and Spain) consistently had values more than 0.5 SD away from the 2007 WHO growth reference median. The third group consisted of countries that could not easily be categorized in one of these two groups, because the mean BMI/A Z-score value of boys was more than 0.5 SD while the value of girls was less than 0.5 SD away from the median of the growth reference population (the former Yugoslav Republic of Macedonia) or the values differed across age groups (Ireland and Slovenia).

#### Prevalence of stunting, underweight and thinness

Stunting, underweight and thinness were rare in all countries. Most values for severe stunting, severe underweight and severe thinness were below 0.2%. All values for stunting and underweight and most values for thinness were below 2.3%. Values for thinness greater than 2.3%, but still close to what is expected in a normally distributed population, were found in Lithuanian 9-year-old girls (3.2%), Slovenian 6-year-old boys (3.1%), Slovenian 7-year-old boys (2.9%) and girls (2.4%) and 7-year-old boys and girls (both 2.5%) of the former Yugoslav Republic of Macedonia.

#### Prevalence of overweight and obesity

Table 4 presents the proportions of overweight and obese boys and girls in each age group and country, based on both the WHO and IOTF definitions. In the six countries with a mean BMI/A Z-score value between the expected reference median value and 0.5 SD away from this value, the prevalence of overweight (including obesity and based on WHO definitions) varied from 18% to 29% (IOTF: 11 − 19%) in boys and from 18% to 28% (IOTF: 15 − 24%) in girls, and the prevalence of obesity varied from 6% to 14% (IOTF: 3 − 7%) in boys and from 5% to 10% (IOTF: 4 − 8%) in girls. In the four countries with BMI/A Z-score values more than 0.5 SD away from the 2007 WHO growth reference median, the prevalence of overweight varied from 32% to 57% (IOTF: 23 − 45%) in boys and from 35% to 50% (IOTF: 30 − 42%) in girls, whereas the prevalence of obesity varied from 14% to 31% (IOTF: 8 − 15%) in boys and from 12% to 21% (IOTF: 8 − 15%) in girls.

**Table 4:** **Prevalence of overweight (including obesity) and obesity in boys and girls aged 6–9 years in COSI Round 2 (2009/2010), by age and country**

Age group and country ^‡^	Prevalence of overweight (including obesity)				Prevalence of obesity			
	WHO definition ^#^		IOTF definition ^*^		WHO definition ^#^		IOTF definition ^*^	
	Boys	Girls	Boys	Girls	Boys	Girls	Boys	Girls
	%							
6-year-olds°	†	†	†	†	†	†	†	†
BEL	18.0^a^	18.2^a^	10.8^a^	15.1^a^	5.8^a^	5.2^a^	2.8^a^	4.2^a^
SVN	23.5^b^	21.7^b^	16.5^b^	18.3^b^	10.0^b^	6.8^b^	5.2^b^	5.8^b^
ESP	38.2^c^	34.5^c^	25.6^c^	30.0^c^	15.1^c^	12.8^c^	8.7^c^	9.9^c^
7-year-olds°°	†	†	†	†	†	†	†	†
BEL	23.1^a^	24.1^a^	15.4^a^	20.3^a^	9.5^a^	8.5^ab^	5.1^a^	6.8^ab^
CZE	24.4^ab^	23.7^a^	17.5^ab^	19.3^a^	10.7^ab^	7.3^ab^	5.0^a^	5.9^ab^
GRC	48.9^c^	44.8^b^	38.1^c^	39.9^b^	23.9^c^	18.6^c^	13.6^b^	14.3^c^
HUN	25.1^ad^	28.2^ac^	18.9^abd^	23.9^ac^	14.2^abde^	10.3^abd^	6.7^ac^	8.2^abc^
IRL	25.7^ad^	30.0^acd^	15.7^ab^	24.9^ac^	8.6^ab^	6.9^ab^	4.1^a^	4.7^a^
LVA	24.5^ae^	22.2^a^	15.9^ab^	17.8^a^	10.8^ab^	7.5^ab^	5.2^a^	5.3^a^
LTU	24.4^ae^	21.0^a^	16.0^ab^	17.7^a^	9.5^ab^	7.1^a^	5.0^a^	5.6^a^
PRT	31.5^bde^	36.2^bcd^	22.7^bd^	30.5^cd^	14.2^abcde^	12.2^abcd^	7.9^ac^	9.6^abc^
SVN	29.6^bde^	24.8^a^	21.1^bd^	21.2^a^	13.5^be^	9.4^ab^	7.1^ac^	6.7^ab^
ESP	44.6^c^	40.4^bd^	33.5^ce^	36.6^bd^	21.0^cd^	14.7^cd^	11.5^bc^	10.5^bc^
MKD	34.0^d^	27.4^a^	26.2^de^	22.9^ac^	18.2^de^	12.1^bd^	10.9^c^	9.6^abc^
8-year-olds°°	†	†	†	†	†	†	†	†
BEL	21.9^a^	22.4^a^	14.2^a^	17.8^a^	7.9^a^	6.7^a^	3.6^a^	4.6^a^
ITA	44.8^b^	40.4^b^	33.9^b^	34.4^b^	22.8^b^	16.0^b^	11.6^b^	11.3^b^
NOR	29.2^c^	26.2^a^	18.6^c^	21.5^c^	11.6^c^	6.2^a^	4.9^a^	3.5^a^
SVN	36.1^d^	32.0^c^	26.5^d^	26.6^d^	17.6^d^	11.9^bc^	8.5^c^	8.5^c^
ESP	45.3^b^	41.0^b^	33.0^b^	34.4^b^	20.0^bd^	14.7^c^	8.3^c^	9.2^bc^
9-year-olds°°°	†	†	†	†	†	†	†	†
BEL	27.3^a^	26.6^a^	18.7^a^	21.8^a^	11.2^a^	9.0^a^	5.1^a^	5.9^a^
GRC	57.2^b^	50.0^b^	45.1^b^	42.3^b^	30.5^b^	20.8^b^	14.7^b^	14.6^b^
IRL	32.2^ac^	30.3^ac^	19.7^ac^	23.1^acd^	10.3^a^	6.8^ac^	4.5^ac^	4.8^ac^
ITA	43.8^d^	37.4^cd^	32.9^d^	31.4^e^	21.9^c^	13.0^d^	10.1^d^	8.4^d^
LTU	27.3^a^	21.3^e^	17.8^a^	16.9^c^	11.3^a^	5.9^c^	4.7^a^	4.1^a^
SVN	36.8^c^	33.6^cd^	25.7^c^	29.1^de^	17.0^d^	13.6^d^	8.0^acd^	9.8^bcd^
ESP	48.9^d^	42.2^bd^	34.1^d^	35.4^be^	22.3^cd^	14.6^d^	9.5^cd^	10.0^bd^

*Abbreviations:* *BMI* body mass index, *BMI/A* BMI-for-age, *COSI* Childhood Obesity Surveillance Initiative, *IOTF* International Obesity Task Force, *WHO* World Health Organization.

^‡^The country codes refer to the International Organization for Standardization (ISO) 3166–1 Alpha-3 country codes and countries were listed in alphabetical order by their full names: BEL, Belgium (Flanders); CZE, Czech Republic; GRC, Greece; HUN, Hungary; IRL, Ireland; ITA, Italy; LVA, Latvia; LTU, Lithuania; NOR, Norway; PRT, Portugal (all regions except Madeira); SVN, Slovenia; ESP, Spain; MKD, the former Yugoslav Republic of Macedonia.

^#^Prevalence estimates were based on the 2007 WHO recommended growth reference for school-age children and adolescents [17]. Body weight was adjusted for clothes worn when measured and children with a BMI/A Z-score < −5 or > +5 were excluded. Overweight and obesity were defined as the proportion of children with a BMI/A value above +1 Z-score and above +2 Z-scores, respectively.

^*^Prevalence estimates were based on the IOTF recommended growth reference for school-age children and adolescents [19]. Body weight was adjusted for clothes worn when measured and children with a BMI/A Z-score < −5 or > +5 were excluded (based on the 2007 WHO growth reference [17]). Overweight and obesity were defined by using cut-off points for BMI, passing through 25 kg/m^2^ and 30 kg/m^2^ by the age of 18 years, respectively.

†Statistically significant difference of proportions across countries for the indicated age group (Chi-squared test; *P* < 0.001).

°Statistically significant difference of proportions between boys and girls for overweight (based on IOTF definition only) and for obesity (based on WHO and IOTF definitions) (Chi-squared test; *P* < 0.001).

°°Statistically significant difference of proportions between boys and girls for overweight and obesity (based on WHO and IOTF definitions) (Chi-squared test; *P* < 0.05).

°°°Statistically significant difference of proportions between boys and girls for overweight (based on WHO and IOTF definitions) and for obesity (based on WHO definitions only) (Chi-squared test; *P* < 0.05).

^a,b,c,d,e^Within each sex-age group (e.g. 6-year-old boys), proportions that share the same superscript letter do not statistically significantly differ from each other (Marascuilo procedure). For example, for the 6-year-old boys and girls, each overweight or obesity prevalence estimate is significantly different from the other two.

The chi-squared test comparing the prevalence estimates across countries was significant for all age groups (*P* < 0.001 for boys and girls separately). The Marascuilo procedure was then used to study country differences for each age group (see Table 4; countries within each sex-age group that share the same superscript letter do not statistically significantly differ from each other).

**Figure 1:**
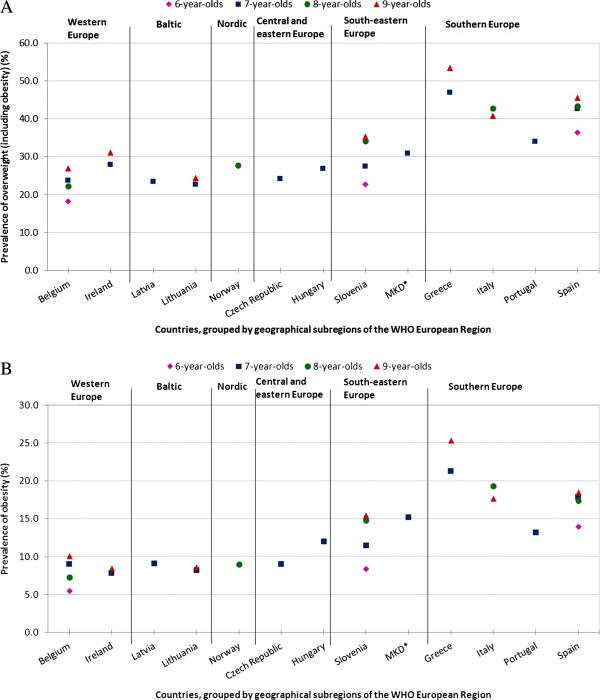
**Geographical distribution of the prevalence of overweight (including obesity) (1A) and obesity (1B) in children aged 6–9 years (sexes combined) of COSI Round 2 (2009/2010), based on WHO definitions** ^**‡**^ **.** Abbreviations: COSI, Childhood Obesity Surveillance Initiative; MKD, the former Yugoslav Republic of Macedonia; WHO, World Health Organization. ^‡^Prevalence estimates were based on the 2007 WHO recommended growth reference for school-age children and adolescents [17]. Children with a body mass index-for-age Z-score < −5 or > +5 were excluded. Belgium represents Flanders only and Portugal represents all regions except Madeira. WHO European Member States are grouped into eight geographic subregions to facilitate comparative analysis and interpretation. None of the central Asian republics or the Commonwealth of Independent States participated in Round 2, and thus these two subregions are excluded from both figures. ^*^MKD is the International Organization for Standardization (ISO) 3166–1 Alpha-3 country code for the former Yugoslav Republic of Macedonia.

Based on WHO definitions, the observed linear increasing trend in the prevalence of overweight with increasing age was significant for Belgian boys and girls (both *P* < 0.001), Greek boys (*P* < 0.001) and girls (*P* < 0.01), Irish boys (*P* < 0.05), Slovenian boys and girls (both *P* < 0.001) and Spanish boys (*P* < 0.001) and girls (*P* < 0.01). The increasing obesity trend with increasing age was significant in Belgian boys and girls (both *P* < 0.001), Greek boys (*P* < 0.001), Slovenian boys and girls (both *P* < 0.001) and Spanish boys (*P* = 0.001). The observed decreasing trend in Italy with increasing age was significant for overweight and obesity in girls (*P* < 0.001). Fewer statistically significant results were found when performing the analyses based on IOTF definitions.Figure 1 illustrates the geographical distribution of the prevalence of overweight and obesity found in Round 2 in 13 countries, grouped by subregions, of the WHO European Region (sexes combined and based on WHO definitions).

### Changes from COSI Round 1 (2007/2008) to COSI Round 2 (2009/2010)

The absolute change in mean values of weight, height and BMI from Round 1 to Round 2 is presented in Table 5 and the absolute change in mean values of W/A, H/A and BMI/A Z-score in Table 6. The direction (increase, decrease or no change) and magnitude of this change differed by country, which is shown by the results of the two-way ANOVA with interaction (country*round). This analysis showed statistically significant results for almost all mean values of BMI (Table 5) and BMI/A Z-score (Table 6). Two-way ANOVA using the additive model was then applied for the mean values with no statistical significant interaction effect. The main effect of country (*P* < 0.0001) on all these values except for weight in 9-year-old girls were statistically significant. The main effect of data collection round (*P* < 0.05) on all weight, BMI and BMI/A Z-score values as well as on one W/A Z-score (6-year-old boys) and some H/A Z-score (7-year-old boys and 9-year-old girls) values were statistically significant. The unpaired t-test showed statistically significant difference in the change in mean values between the two rounds in some age-sex groups in some countries. When this difference was statistically significant for both boys and girls, the direction of the change was the same for both sexes.

**Table 5:** **Absolute change in mean values of weight, height and BMI of boys and girls aged 6–9 years from COSI Round 1 (2007/2008) to COSI Round 2 (2009/2010), by age and country**

Age group and country ^‡^	Weight ^¶^ (kg)		Height ^*^ (cm)		BMI ^#^ (kg/m ^2^ )	
	Boys	Girls	Boys	Girls	Boys	Girls
6-year-olds^§^	*NS*	*NS*	*P < 0.01*	*P < 0.0001*	*P < 0.05*	*P < 0.01*
BEL	- 0.2°°°°	- 0.1°°°°	- 0.2°°°	- 0.2°°°°	- 0.1°°°°	0
SVN	- 0.1	- 0.2	+ 0.4°	+ 0.6°°	- 0.2°°	- 0.3°°°
7-year-olds^§^	*P < 0.05*	*P < 0.01*	*P < 0.001*	*P = 0.0001*	*P < 0.01*	*P < 0.05*
BEL	- 0.1	- 0.4°°°°	- 0.3°°	- 0.6°°°°	0	- 0.1
CZE	+ 0.2	+ 0.4	- 0.2	- 0.2	+ 0.1	+ 0.3
IRL	- 0.7°°	- 0.9°°	- 1.0°°°	- 1.0°°°	- 0.2	- 0.3°
LVA	+ 0.2	+ 0.2	+ 0.2	- 0.1	0	+ 0.1
LTU	0	0	+ 0.3	0	- 0.1	0
PRT	- 0.4	+ 0.1	+ 0.5	+ 0.4	- 0.4°°	0
SVN	- 0.4°	- 0.3	0	0	- 0.2°°	- 0.2°
8-year-olds^§^	*P < 0.0001*	*P < 0.05*	*NS*	*P < 0.05*	*P < 0.0001*	*P < 0.05*
BEL	- 0.1°	- 0.1°	- 0.1°	- 0.1°	0	0
ITA	- 0.6°°°°	- 0.1	+ 0.1	+ 0.3°	- 0.4°°°°	- 0.1
NOR	+ 0.6°°	+ 0.4	- 0.1	0	+ 0.3°°°	+ 0.2°
SVN	+ 0.3	+ 0.3	+ 0.3	+ 0.2	+ 0.1	+ 0.1
9-year-olds^§^	*NS*	*NS*	*P < 0.05*	*P < 0.05*	*P < 0.01*	*NS*
BEL	- 0.3°°	- 0.4°°°	- 0.4°°°°	- 0.4°°°	0	- 0.1°°
ITA	- 0.5°	- 0.5°	+ 0.1	+ 0.1	- 0.3°°	- 0.3°°

*Abbreviations:* *ANOVA* analysis of variance, *BMI* body mass index, *COSI* Childhood Obesity Surveillance Initiative, *BMI/A* BMI-for-age, *H/A* height-for-age, *NS* not significant, *W/A* weight-for-age.

^‡^The country codes refer to the International Organization for Standardization (ISO) 3166–1 Alpha-3 country codes and countries were listed in alphabetical order by their full names: BEL, Belgium (Flanders); CZE, Czech Republic; IRL, Ireland; ITA, Italy; LVA, Latvia; LTU, Lithuania; NOR, Norway; PRT, Portugal (all regions except Madeira); SVN, Slovenia.

^¶^Body weight was adjusted for clothes worn when measured and children with a W/A Z-score < −6 or > +5 were excluded.

^*^Children with a H/A Z-score < −6 or > +6 were excluded.

^#^Body weight was adjusted for clothes worn when measured and children with a BMI/A Z-score < −5 or > +5 were excluded.

^§^Significance level of the two-way ANOVA to assess the interaction effect of country and round on the change in mean values for the indicated age-sex group.

°Statistically significant difference of mean values between the two rounds for the indicated age-sex group (unpaired t-test (equal variance) or unpaired Welch’s t-test (unequal variance); *P* < 0.05).

°°Statistically significant difference of mean values between the two rounds for the indicated age-sex group (unpaired t-test (equal variance) or unpaired Welch’s t-test (unequal variance); *P* < 0.01).

°°°Statistically significant difference of mean values between the two rounds for the indicated age-sex group (unpaired t-test (equal variance) or unpaired Welch’s t-test (unequal variance); *P* ≤ 0.001).

°°°°Statistically significant difference of mean values between the two rounds for the indicated age-sex group (unpaired t-test (equal variance) or unpaired Welch’s t-test (unequal variance); *P* ≤ 0.0001).

**Table 6:** **Absolute change in mean values of weight-for-age, height-for-age and BMI-for-age Z-scores of boys and girls aged 6–9 years from COSI Round 1 (2007/2008) to COSI Round 2 (2009/2010), by age and country**

Age group and country ^‡^	W/A Z-score ^¶^		H/A Z-score ^*^		BMI/A Z-score ^#^	
	Boys	Girls	Boys	Girls	Boys	Girls
6-year-olds^§^	*NS*	*NS*	*NS*	*P < 0.01*	*P < 0.05*	*P < 0.01*
BEL	- 0.02°	- 0.01	+ 0.01	0	- 0.04°°°°	- 0.02°
SVN	- 0.06	- 0.05	+ 0.05	+ 0.10°°	- 0.14°°	- 0.13°°°
7-year-olds^§^	*NS*	*NS*	*NS*	*NS*	*P < 0.001*	*P < 0.05*
BEL	+ 0.02	- 0.02	+ 0.03	- 0.01	0	- 0.03
CZE	+ 0.06	+ 0.09	- 0.02	0	+ 0.09	+ 0.14°
IRL	- 0.03	- 0.04	+ 0.01	+ 0.03	- 0.05	- 0.07
LVA	+ 0.02	0	+ 0.03	- 0.05	- 0.01	+ 0.02
LTU	+ 0.02	- 0.01	+ 0.07°	0	- 0.05	- 0.02
PRT	- 0.09	+ 0.02	+ 0.10°	+ 0.05	- 0.21°°°	- 0.01
SVN	- 0.09°°	- 0.07°	0	+ 0.01	- 0.14°°°	- 0.10°°
8-year-olds^§^	*P = 0.0001*	*NS*	*NS*	*NS*	*P < 0.0001*	*P < 0.05*
BEL	- 0.01	0	0	+ 0.01	- 0.02	- 0.01
ITA	- 0.11°°°	- 0.02	+ 0.02	+ 0.05°	- 0.16°°°°	- 0.05°
NOR	+ 0.10°	+ 0.08	- 0.03	0	+ 0.14°°	+ 0.11°°
SVN	+ 0.01	- 0.01	- 0.01	- 0.04	+ 0.01	0
9-year-olds^§^	*P < 0.05*	*NS*	*NS*	*NS*	*P < 0.01*	*NS*
BEL	0	- 0.01	0	+ 0.03°	- 0.01	- 0.03
ITA	- 0.09°	- 0.06	+ 0.02	+ 0.03	- 0.13°°°	- 0.09°

*Abbreviations:* *ANOVA* analysis of variance, *BMI* body mass index, *COSI* Childhood Obesity Surveillance Initiative, *BMI/A* BMI-for-age, *H/A* height-for-age, *NS* not significantl, *W/A* weight-for-age.

^‡^The country codes refer to the International Organization for Standardization (ISO) 3166–1 Alpha-3 country codes and countries were listed in alphabetical order by their full names: BEL, Belgium (Flanders); CZE, Czech Republic; IRL, Ireland; ITA, Italy; LVA, Latvia; LTU, Lithuania; NOR, Norway; PRT, Portugal (all regions except Madeira); SVN, Slovenia.

^¶^Body weight was adjusted for clothes worn when measured and children with a W/A Z-score < −6 or > +5 were excluded.

^*^Children with a H/A Z-score < −6 or > +6 were excluded.

^#^Body weight was adjusted for clothes worn when measured and children with a BMI/A Z-score < −5 or > +5 were excluded.

^§^Significance level of the two-way ANOVA to assess the interaction effect of country and round on the change in mean values for the indicated age-sex group.

°Statistically significant difference of mean values between the two rounds for the indicated age-sex group (unpaired t-test (equal variance) or unpaired Welch’s t-test (unequal variance); *P* < 0.05).

°°Statistically significant difference of mean values between the two rounds for the indicated age-sex group (unpaired t-test (equal variance) or unpaired Welch’s t-test (unequal variance); *P* < 0.01).

°°°Statistically significant difference of mean values between the two rounds for the indicated age-sex group (unpaired t-test (equal variance) or unpaired Welch’s t-test (unequal variance); *P* ≤ 0.001).

°°°°Statistically significant difference of mean values between the two rounds for the indicated age-sex group (unpaired t-test (equal variance) or unpaired Welch’s t-test (unequal variance); *P* < 0.0001).

The absolute change in overweight and obesity prevalence estimates (based on WHO definitions [17]) from Round 1 to Round 2 is presented in Table 7. The prevalence of overweight (including obesity) statistically significantly decreased in Belgian 6-year-old boys, Italian 8-year-old boys and girls, Italian 9-year-old boys, Portuguese 7-year-old boys, Slovenian 6- and 7-year-old boys and Slovenian 7-year-old girls. A statistically significant absolute increase in overweight prevalence was observed in Latvian 7-year-old girls and Norwegian 8-year-old boys. The prevalence of obesity statistically significantly decreased in Italian 8-year-old boys, Italian 9-year-old boys and girls and Slovenian 7-year-old boys. A statistically significant absolute increase in obesity prevalence was observed in Latvian 7-year-old boys and girls and Norwegian 8-year-old boys.

**Table 7:** **Absolute change in prevalence of overweight (including obesity) and obesity in boys and girls aged 6–9 years from COSI Round 1 (2007/2008) to COSI Round 2 (2009/2010), by age and country**

Age group and country ^‡^	Overweight (including obesity) (%) ^#^		Obesity (%) ^#^	
	Boys	Girls	Boys	Girls
6-year-olds				
BEL	- 1.3°°°	- 0.2	- 0.2	+ 0.1
SVN	- 4.6°°	- 1.9	- 1.6	- 1.6
7-year-olds				
BEL	- 0.4	- 0.1	+ 0.4	+ 0.5
CZE	+ 2.9	+ 3.5	+ 1.0	+ 1.5
IRL	- 1.9	- 1.0	- 0.1	- 2.9
LVA	+ 0.4	+ 3.2°	+ 2.1°	+ 2.9°°°
LTU	- 0.4	0	+ 0.1	- 0.1
PRT	- 9.0°°°°	+ 0.7	- 2.5	- 0.4
SVN	- 3.0°	- 3.2°	- 2.1°	- 0.4
8-year-olds				
BEL	- 0.2	- 0.3	- 0.1	+ 0.4
ITA	- 4.2°°°°	- 2.2°	- 3.9°°°°	- 1.3
NOR	+ 6.2°°°	+ 3.1	+ 4.2°°°	+ 0.2
SVN	+ 0.2	+ 0.3	+ 1.3	+ 1.0
9-year-olds				
BEL	- 0.1	- 0.6	+ 0.4	+ 0.1
ITA	- 3.3°	- 2.7	- 3.8°°	- 2.8°°

*Abbreviations: BMI/A* body mass index-for-age, *COSI* Childhood Obesity Surveillance Initiative, *WHO* World Health Organization.

^‡^The country codes refer to the International Organization for Standardization (ISO) 3166–1 Alpha-3 country codes and countries were listed in alphabetical order by their full names: BEL, Belgium (Flanders); CZE, Czech Republic; IRL, Ireland; ITA, Italy; LVA, Latvia; LTU, Lithuania; NOR, Norway; PRT, Portugal (all regions except Madeira); SVN, Slovenia.

^#^Prevalence estimates were based on the 2007 WHO recommended growth reference for school-age children and adolescents [17]. Body weight was adjusted for clothes worn when measured and children with a BMI/A Z-score < −5 or > + 5 were excluded. Overweight and obesity were defined as the proportion of children with a BMI/A value above +1 Z-score and above + 2 Z-scores, respectively.

°Statistically significant difference of proportions between the two rounds for the indicated age-sex group (*z* test; *P* < 0.05).

°°Statistically significant difference of proportions between the two rounds for the indicated age-sex group (*z* test; *P* < 0.01).

°°°Statistically significant difference of proportions between the two rounds for the indicated age-sex group (*z* test; *P* < 0.001).

°°°°Statistically significant difference of proportions between the two rounds for the indicated age-sex group (*z* test; *P* ≤ 0.0001).

## Discussion

### COSI Round 2 (2009/2010)

When adjusting weight (Table 1) and height (Table 2) for age, positive W/A and H/A Z-score values were found in all countries. This means that the country values were higher than the population median values of the 2007 WHO growth reference [17] and thus the COSI children were heavier and taller than the reference population.

All country mean BMI/A Z-score values (Table 3) were positive − thus higher than the 2007 WHO growth reference population [17] − but varied largely among the countries. Grouping of countries on the basis of having or not having a mean value of 0.5 SD away from the reference median suggests the presence of a north–south gradient with the highest BMI/A Z-score values found in southern European countries. Categorization of countries based on prevalence estimates (regardless of WHO or IOTF definitions) showed similar groups of countries. These findings also suggest the presence of a north–south gradient with the highest overweight and obesity prevalence estimates noted in southern European countries. A north–south gradient was also observed in COSI Round 1 [5] as well as in other European-wide studies carried out among adolescents in 2010 [23, 24].

Possible explanations for the apparent north–south gradient remain unclear. Studies carried out in European children [25] and adolescents [26] suggest that shortness (low H/A) might be one of the explanations. The mean H/A Z-score values found in Italy, Portugal and Spain (but not those in Greece) were indeed significantly lower (but still higher than the growth reference values) than the values of almost all other countries. Other suggested explanations concern, among others, birth weight [27, 28], sleep duration [29], dietary [23] or physical activity patterns [23], which seem to vary among children by country or subregion in Europe as well. To what extent these variables can explain the overweight north–south gradient found in COSI surveys would need to be explored further. Information was collected by some COSI countries and will be published elsewhere.

Stunting, thinness and underweight were rare among the 6 − 9-year-olds in any of the COSI countries. It is, however, uncertain whether these results found in the COSI countries can be generalized to the entire WHO European Region because no comparable studies in other countries could be found that collected data on these indicators for the same age range in 2009 or later. But studies done among younger children (0 − 5-year-olds) showed that stunting is considerable in some countries in the WHO European Region (Armenia, 19.3% overall and 36.5% in Syunik [30]; Bosnia and Herzegovina, 8.9% overall and 9.9% in the Federation of Bosnia and Herzegovina [31]; Kazakhstan, 13.1% overall and 36.2% in Aktobe Oblast [32]; Serbia, 6.6% overall and 8.3% in Belgrade [33]; and the former Yugoslav Republic of Macedonia, 4.9% overall and 13.3% in southwest region [34] (based on measured weight and height and the 2006 WHO child growth standards [35]).

### Changes from COSI Round 1 (2007/2008) to Round 2 (2009/2010)

In two years, the change within country in mean weight varied from a decrease of −0.9 kg to an increase of +0.6 kg and the change in mean height varied from a decrease of −1.0 cm to an increase of +0.6 cm (Table 5). The observed range of the country weight and height differences is nevertheless plausible. No statistically significant variation in the change in mean W/A Z-score and H/A Z-score values was found across the countries in most age groups (Table 6), which suggest the presence of the same weight and height (adjusted for age) developments in the COSI countries. This was not the case for BMI.

The absolute change in mean BMI ranged from a statistically significant decrease of −0.4 kg/m^2^ (Italy and Portugal) to a statistically significant increase of +0.3 kg/m^2^ (Norway). There was variation in the change in mean BMI across the countries in the 6-, 7- and 8-year-old age groups and the variation remained statistically significant when BMI was adjusted for age (Table 6). A significant decrease of 0.10 BMI/A Z-score per year was observed in Portuguese boys. Interestingly, a statistically significant decrease was observed in southern European countries with higher absolute BMI/A Z-scores (Italy, Portugal and Slovenia) and an increase in the group of countries with lower absolute BMI/A Z-score values (Czech Republic and Norway). Countries with higher prevalence of overweight in Round 1 (e.g. Italy and Portugal) [5] showed a decrease in prevalence (but still had among the highest estimates in Round 2), and the country with lower prevalence in Round 1 (e.g. Latvia and Norway) [5] showed an increase in prevalence (but still had a lower estimate than the countries that showed the highest decrease from Round 1 to Round 2). Changes could only be assessed for nine COSI countries and the statistically significant results suggest different developments in these countries, hence, a conclusion about the overall pattern (increase or decrease) for the entire WHO European Region (53 Member States) cannot be drawn.

### Strengths

COSI requires the inclusion of national samples (unless a country’s political system is decentralized like, for instance, in Belgium), and the children are selected from the school population, which is presumed to be representative of the total population for the 6-, 7-, 8- and 9-year-olds.

COSI provides a unique large dataset with a total number of more than 168 000 children with valid measurements in Round 1 [5] and about 220 000 children in Round 2. It is expected that the number of children will increase in each future COSI round because four new countries (Albania, Republic of Moldova, Romania and Turkey) participated in the third COSI data collection round (2012/2013) and other countries may follow by joining the fourth round during the school year 2015/2016.

The standardized weight and height measurements in a large number of countries and the application of a consistent data collection protocol enabled the use of multiple comparisons (Tables 1, 2, 3 and 4), and the repeated COSI rounds made it possible to assess the changes over time (Tables 5, 6 and 7).

### Limitations

Some differences in sample size achieved might have influenced the results. Seven countries in Round 2 did not obtain a final sample of children that contained more than 60% of the approached children and fell within the targeted age group, while eight countries did not achieve the minimum final effective sample size of ≈ 2800 children per age group (see Additional file 2). However, Table 6 showed that all difference values of BMI/A Z-score higher than 0.05 per year were already statistically significant, which suggest that the COSI data have sufficient power to detect a significant difference of 0.10 per year if it really existed (on which sample size calculation for COSI was based – see Methods section). This also means that it is almost certain that a real change of 0.10 BMI/A Z-score per year did not happen in the COSI countries where no statistically significant changes were found.

The analyses were performed unweighted, because sampling weights to adjust for the applied sampling design, oversampling and non-response rate were available for only three countries in Round 1 [5] and four countries in Round 2. This was mainly due to the incomplete registration of all children in the schools and classes. We do not know the effect of the unweighted analyses on the results, but we would expect this to be limited due to the nationally representative sampling of children.

In an optimal study design, the same scales and stadiometers should be used with similar and adequate calibration procedures. For the majority of the countries, the same equipment was used throughout the country, but data comparability would have been improved if identical equipment would have been used by each country. This was not set as mandatory in the COSI protocol, largely because of cost implications. The monitoring of data quality procedures, however, was stressed throughout the measurement period.

## Conclusions

The WHO COSI includes repeated data collection rounds in 2 − 3-year intervals in order to assess changes in weight, height and BMI as well as in overweight and obesity prevalence estimates. The results show that with the present COSI data it is possible to detect relevant changes between rounds. A period of two years, with just two rounds of data collection, is, however, inadequate to identify clear trends within countries. Hence, continuation of the surveys will be important to evaluate the currently observed changes over a longer period. These changes varied significantly across the countries and showed little decline. All countries will thus need to strengthen their efforts in order to become on track with achieving the European Charter’s goal to reverse the obesity epidemic by 2015 [1]. Furthermore, it seems that active implementation of policies or interventions to counteract overweight and obesity have been triggered more by the countries with higher values in Round 1 than by the countries with lower values. It could be that the latter group of countries did not see the urgency to keep their values stable and thus to introduce overweight preventive interventions targeting school-aged children for this purpose.

## Electronic supplementary material

Additional file 1: **Sampling characteristics for each of the thirteen countries that participated in COSI Round 2 (2009/2010).** (DOCX 59 KB)

Additional file 2: **Number of children targeted, measured and included in the final dataset for each of the thirteen countries that participated in COSI Round 2 (2009/2010).** (DOCX 59 KB)

Additional file 3: **Implementation of the WHO European Childhood Obesity Surveillance Initiative’s protocol characteristics by each of the thirteen countries that participated in COSI Round 2 (2009/2010).** (DOCX 59 KB)

Additional file 4: **Ethics approval procedures for participation in the WHO European Childhood Obesity Surveillance Initiative applied by each of the thirteen countries that participated in COSI Rounds 1 (2007/2008) and 2 (2009/2010).** (DOCX 60 KB)

Additional file 5: **BMI cut-off values for overweight and obesity for children aged 6–9 years, by sex and age, according to WHO and IOTF definitions.** (DOCX 62 KB)

Additional file 6: **Median and interquartile range (Q1–Q3) values of weight and BMI of boys and girls aged 6–9 years in COSI Round 2 (2009/2010), by age and country.** (DOCX 62 KB)
