# Supplementary material for: WHO European Childhood Obesity Surveillance Initiative: body mass index and level of overweight among 6–9-year-old children from school year 2007/2008 to school year 2009/2010
Source: BMC Public Health. 2014 Aug 7;14:806. doi: 10.1186/1471-2458-14-806 (PMC4289284; doi:10.1186/1471-2458-14-806)
Supplement: Supplementary file 1 — Additional file 1: Sampling characteristics for each of the thirteen countries that participated in COSI Round 2 (2009/2010). (DOCX 59 KB) [file 12889_2014_6942_MOESM1_ESM.docx]

**Additional file 1** Sampling characteristics for each of the thirteen countries that participated in COSI Round 2 (2009/2010)

| Characteristics | Countries^a^ | | | | | | | | | | | | |
| --- | --- | --- | --- | --- | --- | --- | --- | --- | --- | --- | --- | --- | --- |
|  | BEL | CZE | GRC | HUN | IRL | ITA | LVA | LTU | NOR | PRT | SVN | ESP | MKD |
| Sampling design |  |  |  |  |  |  |  |  |  |  |  |  |  |
| Inclusion of entire targeted age group | √ |  |  |  |  |  |  |  |  |  |  |  |  |
| Cluster sampling design |  | √ | √ | √ | √ | √ | √ | √ | √ | √ | √ | √ | √ |
| New sample of schools | NA | √^b^ | √ | √ |  | √ | √ |  |  |  | √ | √ | √ |
| Same schools as selected in COSI Round 1 | NA |  |  |  | √ |  |  | √ | √ | √ |  |  |  |
| Schools (PSU) |  |  |  |  |  |  |  |  |  |  |  |  |  |
| Total approached (n) | NA | 85^b^ | 150 | 164 | 192 | NA | 174 | 164 | 131^c^ | 185 | 167 | 163 | 115 |
| Total included (n) | NA | 67^b^ | 123 | 98 | 154 | NA | 169 | 162 | 125^c^ | 172 | 167 | 144 | 100 |
| Participation rate (%) | NA | 78.8 | 82.0 | 59.8 | 80.2 | NA | 97.1 | 98.8 | 95.4 | 93.0 | 100 | 88.3 | 87.0 |
| Classes (SSU) |  |  |  |  |  |  |  |  |  |  |  |  |  |
| Total approached (n) | NA | NA | 337 | 346 | 328 | 2437^d^ | 279 | 604 | 131^e^ | 372 | 950 | 594 | 221 |
| Total included (n) | NA | NA | 265 | 167 | 260 | 2437^d^ | 267 | 604 | 125^e^ | 318 | 950 | 594 | 210 |
| Participation rate (%) | NA | NA | 78.6 | 48.3 | 79.3 | 100 | 95.7 | 100 | 95.4 | 85.5 | 100 | 100 | 95.0 |

Abbreviations: COSI, Childhood Obesity Surveillance Initiative; NA, not applicable; √, applicable; PSU, primary sampling unit; SSU, secondary sampling unit.

^a^The country codes refer to the International Organization for Standardization (ISO) 3166-1 Alpha-3 country codes and countries were listed in alphabetical order by their full names: BEL, Belgium (Flanders); CZE, Czech Republic; GRC, Greece; HUN, Hungary; IRL, Ireland; ITA, Italy; LVA, Latvia; LTU, Lithuania; NOR, Norway; PRT, Portugal (all regions except Madeira); SVN, Slovenia; ESP, Spain; MKD, the former Yugoslav Republic of Macedonia.

^b^Paediatric clinics formed the PSU.

^c^Counties formed the PSU.

^d^Classes formed the PSU.

^e^Schools formed the SSU.
