# Supplementary material for: WHO European Childhood Obesity Surveillance Initiative: body mass index and level of overweight among 6–9-year-old children from school year 2007/2008 to school year 2009/2010
Source: BMC Public Health. 2014 Aug 7;14:806. doi: 10.1186/1471-2458-14-806 (PMC4289284; doi:10.1186/1471-2458-14-806)
Supplement: Supplementary file 2 — Additional file 2: Number of children targeted, measured and included in the final dataset for each of the thirteen countries that participated in COSI Round 2 (2009/2010). (DOCX 59 KB) [file 12889_2014_6942_MOESM2_ESM.docx]

**Additional file 2** Number of children targeted, measured and included in the final dataset for each of the thirteen countries that participated in COSI Round 2 (2009/2010)

|  | Countries^a^ | | | | | | | | | | | | | |
| --- | --- | --- | --- | --- | --- | --- | --- | --- | --- | --- | --- | --- | --- | --- |
|  | BEL | CZE | GRC | HUN | IRL | ITA | LVA | LTU | NOR | PRT | SVN | ESP | MKD |  |
| Total number of children sampled and |  |  |  |  |  |  |  |  |  |  |  |  |  |  |
| invited to participate (n) | 267087 | 2595 | 7432 | ND | 6293 | 46734 | 5221 | 12021 | 3647 | 4725 | 19431 | 12886 | 2940 |  |
| measured (%) | 99.8 | 94.1^b^ | 76.5 | ND | 64.0 | 91.0 | 82.1 | 81.5 | 87.3 | 79.1 | 82.2 | 59.4 | 96.7 |  |
| with complete information (%) | 49.9 | 94.1^b^ | 76.5 | ND | 63.9 | 89.9 | 82.1 | 81.5 | 87.0 | 79.1 | 82.2 | 59.4 | 96.7 |  |
| that fell within the targeted age group(s) (%) | 49.9 | 49.0^b^ | 70.9 | ND | 31.6 | 89.2 | 54.4 | 55.9 | 71.9 | 38.4 | 82.0 | 59.4 | 93.3 |  |
| Total number of targeted children included for this paper’s analyses (n) | 133156 | 1271 | 5269 | 1235 | 1986 | 41672 | 2838 | 6721 | 2621 | 1813 | 15938 | 7656 | 2744 |  |
| 6-year-olds; boys/girls (n) | 26542/ 26105 | 0 | 0 | 0 | 0 | 0 | 0 | 0 | 0 | 0 | 1801/ 1834 | 901/ 917 | 0 |  |
| 7-year-olds; boys/girls (n) | 7804/ 6841 | 638/ 633 | 1293/ 1259 | 553/ 682 | 534/ 466 | 0 | 1381/ 1457 | 1648/ 1794 | 0 | 910/ 903 | 2759/ 2493 | 1106/ 1101 | 1429/ 1315 |  |
| 8-year-olds; boys/girls (n) | 23632/ 23171 | 0 | 0 | 0 | 0 | 13197/ 12812 | 0 | 0 | 1335/ 1286 | 0 | 2690/ 2599 | 1083/ 1047 | 0 |  |
| 9-year-olds; boys/girls (n) | 9805/ 9256 | 0 | 1288/ 1429 | 0 | 488/ 498 | 8280/ 7383 | 0 | 1659/ 1620 | 0 | 0 | 952/ 810 | 749/ 752 | 0 |  |

Abbreviations: COSI, Childhood Obesity Surveillance Initiative; ND, not determined.

^a^The country codes refer to the International Organization for Standardization (ISO) 3166-1 Alpha-3 country codes and countries were listed in alphabetical order by their full names: BEL, Belgium (Flanders); CZE, Czech Republic; GRC, Greece; HUN, Hungary; IRL, Ireland; ITA, Italy; LVA, Latvia; LTU, Lithuania; NOR, Norway; PRT, Portugal (all regions except Madeira); SVN, Slovenia; ESP, Spain; MKD, the former Yugoslav Republic of Macedonia.

^b^Data collected from October 2009 to December 2009 and from January 2011 to April 2011 were not taken into account in this paper.
