# Supplementary material for: WHO European Childhood Obesity Surveillance Initiative: body mass index and level of overweight among 6–9-year-old children from school year 2007/2008 to school year 2009/2010
Source: BMC Public Health. 2014 Aug 7;14:806. doi: 10.1186/1471-2458-14-806 (PMC4289284; doi:10.1186/1471-2458-14-806)
Supplement: Supplementary file 3 — Additional file 3: Implementation of the WHO European Childhood Obesity Surveillance Initiative’s protocol characteristics by each of the thirteen countries that participated in COSI Round 2 (2009/2010). (DOCX 59 KB) [file 12889_2014_6942_MOESM3_ESM.docx]

**Additional file 3** Implementation of the WHO European Childhood Obesity Surveillance Initiative’s protocol characteristics by each of the thirteen countries that participated in COSI Round 2 (2009/2010)

| COSI protocol characteristics | Countries^a^ | | | | | | | | | | | | |
| --- | --- | --- | --- | --- | --- | --- | --- | --- | --- | --- | --- | --- | --- |
|  | BEL | CZE | GRC | HUN | IRL | ITA | LVA | LTU | NOR | PRT | SVN | ESP | MKD |
| COSI surveillance system |  |  |  |  |  |  |  |  |  |  |  |  |  |
| Integration with routine measurements | √ | √ |  | √ |  |  |  |  |  |  | √ |  |  |
| Newly established surveillance system |  |  | √ |  | √ | √ | √ | √ | √ | √ |  | √ | √ |
| Data collection period |  |  |  |  |  |  |  |  |  |  |  |  |  |
| Starting (month/year) | 09/09 | 01/10^b^ | 11/10 | 04/10 | 10/10 | 04/10 | 03/10 | 02/10 | 09/10 | 04/10 | 04/10 | 10/10 | 10/10 |
| Ending (month/year) | 08/10 | 12/10^b^ | 03/11 | 06/10 | 11/10 | 10/10 | 04/10 | 05/10 | 12/10 | 12/10 | 04/10 | 05/11 | 12/10 |
| Informed parental consent approach |  |  |  |  |  |  |  |  |  |  |  |  |  |
| Passive | NA^c^ |  |  |  |  | √ | √ |  |  |  |  |  |  |
| Active | NA^c^ | √ | √ | √ | √ |  |  | √ | √ | √ | √ | √ | √ |
| Field examiners |  |  |  |  |  |  |  |  |  |  |  |  |  |
| External health professionals  linked to the school |  | √ |  | √ |  | √ |  |  | √ |  |  |  |  |
| Physical education teachers |  |  |  |  |  |  |  |  |  |  | √ |  |  |
| Nationally or regionally based  examiners | √ |  | √ |  | √ |  | √ | √ |  | √ |  | √ | √ |

Abbreviations: COSI, Childhood Obesity Surveillance Initiative; NA, not applicable; √, applicable.

^a^The country codes refer to the International Organization for Standardization (ISO) 3166-1 Alpha-3 country codes and countries were listed in alphabetical order by their full names: BEL, Belgium (Flanders); CZE, Czech Republic; GRC, Greece; HUN, Hungary; IRL, Ireland; ITA, Italy; LVA, Latvia; LTU, Lithuania; NOR, Norway; PRT, Portugal (all regions except Madeira); SVN, Slovenia; ESP, Spain; MKD, the former Yugoslav Republic of Macedonia.

^b^ Data collected from October 2009 to December 2009 and from January 2011 to April 2011 were not taken into account in this paper.

^c^In Flanders (Belgium), measurements of body weight and body height in schoolchildren are part of the preventive medical examinations, which are carried out within the school system and are mandatory by law.
