# Supplementary material for: WHO European Childhood Obesity Surveillance Initiative: body mass index and level of overweight among 6–9-year-old children from school year 2007/2008 to school year 2009/2010
Source: BMC Public Health. 2014 Aug 7;14:806. doi: 10.1186/1471-2458-14-806 (PMC4289284; doi:10.1186/1471-2458-14-806)
Supplement: Supplementary file 4 — Additional file 4: Ethics approval procedures for participation in the WHO European Childhood Obesity Surveillance Initiative applied by each of the thirteen countries that participated in COSI Rounds 1 (2007/2008) and 2 (2009/2010). (DOCX 60 KB) [file 12889_2014_6942_MOESM4_ESM.docx]

**Additional file 4** Ethics approval procedures for participation in the WHO European Childhood Obesity Surveillance Initiative applied by each of the thirteen countries that participated in COSI Rounds 1 (2007/2008) and 2 (2009/2010)

**Belgium**

Belgium participated in COSI rounds 1 and 2. In both COSI rounds, ethical approval prior to data collection (weight and height measurements) were not required as the mandatory health check of children aged 3 to 18 years is regulated by law. The Centres for Pupils Counselling (Centra voor Leerlingenbegeleiding) performed the registration, which included the measurement of height, weight, vision, position of the eyes, depth perception, colour vision and pubertal development for boys and girls. Thereafter the Flemish Agency for Care and Health received the data according to Belgian decree.

**Czech Republic**

The Czech Republic participated in COSI rounds 1 and 2. The study was agreed by the Institutional Ethical Committee of the Institute of Endocrinology in Prague after having received the detailed design of the study in 2008. The study protocol was agreed for COSI rounds in 2007/2008, 2009/2010 and any following rounds. The approval letter is archived in the Ethical Committee of the Institute of Endocrinology. Paediatricians who perform the obligatory 7-year preventive check-up at paediatric clinics informed parents about the study and the measuring procedures, and gave them an informed consent form to sign before their child was measured for the study. Children were also asked to express their agreement to participate in the study before being included.

**Greece**

Greece participated in COSI round 2. The survey protocol was submitted by the COSI principal investigator of Greece to the Ethics Committee of the Alexander Technological Educational Institute of Thessaloniki, the institute that is responsible for the implementation of COSI in Greece. After having studied the protocol in detail, the Ethics Committee gave its approval. The members of the Ethics Committee were: Professor Thomais Karagiozoglou, Professor Menelaos Zafrakas and Professor Vassilis Bambidis, all affiliated with the Alexander Technological Educational Institute of Thessaloniki.

**Hungary**

Hungary participated in COSI round 2. Ethical permission for the ‘Hungarian Childhood Study’ in 2010 was granted by the Scientific and Research Ethics Committee of the Medical Research Council (approval reference number 22-272/2007-1018EKU). The entire ethics approval procedure is regulated by Act CLIV of 1997 on Health Affairs. The approval included the purpose of the survey, the study and sampling design, the implementation in schools, the measurement protocol, the procedures for training examiners, project timetable, data management and ethical considerations as well as the information letter that was given to the parents, the written consent form and the questionnaires.

**Ireland**

Ireland participated in COSI rounds 1 and 2. The ‘Surveillance of obesity in Irish school children’ study received ethical approval from the University College Dublin (Dublin, Ireland) Research Ethics Committee (<http://www.ucd.ie/researchethics/>) in 2008 and 2010. The approval included selection and recruitment of children through primary schools, the measurement procedures, obtaining written consent from parents and verbal consent from children, and the text in the information sheet and the consent form to the parents.

**Italy**

Italy participated in COSI rounds 1 and 2. The Institutional Ethical Committee of the Italian National Institute of Health reviewed and approved the protocol of both rounds, including the use of opt-out consent, that is, parents could refuse participation in the study by specifically declining consent, and the lack of a returned form was taken to imply consent for their child’s participation.

**Latvia**

Latvia participated in COSI rounds 1 and 2. Ethics approval was sought for both rounds from the Central Medical Ethics Committee, which is affiliated with the Ministry of Health (<http://www.vm.gov.lv/lv/nozare/centrala_medicinas_etikas_komiteja/>). The information that was required by the Ethics Committee as part of the ethics approval procedure included: the names of the researchers and their curricula vitae, study objectives, a description of the protocol, methods and technologies, expected results, ethical considerations, financing, information on the study subjects (children and parents), the informed consent form, confidentiality, etc.

**Lithuania**

Lithuania participated in COSI rounds 1 and 2. Ethics approval for the ‘Growth surveillance study of Lithuanian children’ was sought in January 2008 (round 1) from the Lithuanian Bioethics Committee. Permission was granted in March 2008. The documents that needed to be sent to the Committee as part of the ethics approval procedures were: the application requesting permission to perform the biomedical survey, a filled out application form for biomedical research, the study protocol, questionnaires and information on the confidentiality of personal information, a filled out ethical evaluation form for biomedical research, the curriculum vitae of the applicant and the applicant’s criminal history. An extension of the duration of the study was requested in 2010 and granted by the Lithuanian Bioethics Committee. In addition, approval to implement the study in schools was sought from the Lithuanian Ministry of Education and Science and the education departments in local municipalities in both rounds.

**Norway**

Norway participated in COSI rounds 1 and 2. Ethics approval for ‘The Norwegian Childhood Study’ was sought for both rounds from the Regional Committee for Medical and Health Research Ethics in 2008 and 2010 (<https://helseforskning.etikkom.no/ikbViewer/page/komiteerogmoter/sorost/sekretariat?p_dim=34981&region=10795>). The approval included permission to implement the study in schools, the measurement procedures, the information that was provided to the parents and the written consent form. The reference number for the ethics approval is S-08181a and the contact person is Professor Jørgen Hardang.

**Portugal**

Portugal participated in COSI rounds 1 and 2. The General Directorate of Health in Lisbon sought ethics approval in both rounds from the Portuguese Data Protection Authority (<http://www.cnpd.pt/bin/legal/forms.htm>). All methodological aspects, particularly the anthropometric measurements of the children and a precise explanation of the informed parental consent and children´s consent on the day of the measurements, were included in the application. The approval letter was sent to the General Directorate of Health and archived there.

**Slovenia**

Slovenia participated in COSI rounds 1 and 2. Data on body weight, height, gender and age were collected within the SLOFit monitoring system, which involves the assessment of growth and motor development in schoolchildren. The SLOFit system has been part of the compulsory school programme in all Slovenian schools for the last 32 years. Ethics approval was not required because data gathering, based on positive written consent of the parents, is regulated within school legislation. This legislation prescribes that the gathered data can be used for research purposes in an anonymized form without ethics approval.

**Spain**

Spain participated in COSI round 2. Ethics approval from a local ethics committee was not required. Instead, the implementation of the study and access to the schools were regulated and approved by the National Health Authority and the Regional Health Authorities.

**The former Yugoslav Republic of Macedonia**

The former Yugoslav Republic of Macedonia participated in COSI round 2. Ethics approval from a local ethics committee was not sought. The COSI measurements were done within the framework of the National Annual Program of Public Health, which is annually adopted and funded by the Government of the former Yugoslav Republic of Macedonia. The Institute for Public Health of the Republic of Macedonia and Centers of Public Health are obliged to perform the measurements annually according to this Program with the objective to obtain data on the nutritional status of children. The measurements were done in public schools, which are owned by the Government.
