# Supplementary material for: WHO European Childhood Obesity Surveillance Initiative: body mass index and level of overweight among 6–9-year-old children from school year 2007/2008 to school year 2009/2010
Source: BMC Public Health. 2014 Aug 7;14:806. doi: 10.1186/1471-2458-14-806 (PMC4289284; doi:10.1186/1471-2458-14-806)
Supplement: Supplementary file 5 — Additional file 5: BMI cut-off values for overweight and obesity for children aged 6–9 years, by sex and age, according to WHO and IOTF definitions. (DOCX 62 KB) [file 12889_2014_6942_MOESM5_ESM.docx]

**Additional file 5** BMI cut-off values for overweight and obesity for children aged 6–9 years, by sex and age, according to WHO and IOTF definitions

|  | Overweight | | | | Obesity | | | |
| --- | --- | --- | --- | --- | --- | --- | --- | --- |
| Age | Boys | | Girls | | Boys | | Girls | |
| years : months | WHO^a^ | IOTF^b^ | WHO^a^ | IOTF^b^ | WHO^c^ | IOTF^d^ | WHO^c^ | IOTF^d^ |
| 6:0 | 16.76 | 17.52 | 17.01 | 17.33 | 18.52 | 19.76 | 19.22 | 19.61 |
| 6:1 | 16.78 | 17.54 | 17.03 | 17.35 | 18.55 | 19.82 | 19.26 | 19.67 |
| 6:2 | 16.80 | 17.56 | 17.05 | 17.37 | 18.59 | 19.88 | 19.31 | 19.72 |
| 6:3 | 16.82 | 17.59 | 17.07 | 17.39 | 18.63 | 19.94 | 19.35 | 19.78 |
| 6:4 | 16.84 | 17.62 | 17.09 | 17.42 | 18.67 | 20.01 | 19.39 | 19.84 |
| 6:5 | 16.86 | 17.64 | 17.11 | 17.45 | 18.70 | 20.08 | 19.44 | 19.90 |
| 6:6 | 16.89 | 17.67 | 17.13 | 17.48 | 18.75 | 20.15 | 19.48 | 19.96 |
| 6:7 | 16.91 | 17.70 | 17.15 | 17.51 | 18.79 | 20.22 | 19.53 | 20.03 |
| 6:8 | 16.94 | 17.73 | 17.18 | 17.54 | 18.83 | 20.29 | 19.58 | 20.10 |
| 6:9 | 16.96 | 17.77 | 17.20 | 17.58 | 18.88 | 20.36 | 19.63 | 20.17 |
| 6:10 | 16.99 | 17.80 | 17.23 | 17.61 | 18.92 | 20.44 | 19.68 | 20.24 |
| 6:11 | 17.02 | 17.84 | 17.26 | 17.65 | 18.97 | 20.51 | 19.73 | 20.32 |
| 7:0 | 17.05 | 17.88 | 17.29 | 17.69 | 19.02 | 20.59 | 19.79 | 20.39 |
| 7:1 | 17.08 | 17.91 | 17.32 | 17.73 | 19.07 | 20.66 | 19.85 | 20.47 |
| 7:2 | 17.11 | 17.95 | 17.35 | 17.78 | 19.12 | 20.74 | 19.90 | 20.55 |
| 7:3 | 17.14 | 17.99 | 17.38 | 17.82 | 19.17 | 20.82 | 19.96 | 20.63 |
| 7:4 | 17.17 | 18.04 | 17.42 | 17.87 | 19.22 | 20.90 | 20.02 | 20.72 |
| 7:5 | 17.20 | 18.08 | 17.45 | 17.91 | 19.27 | 20.98 | 20.09 | 20.80 |
| 7:6 | 17.23 | 18.12 | 17.49 | 17.96 | 19.33 | 21.06 | 20.15 | 20.89 |
| 7:7 | 17.26 | 18.17 | 17.53 | 18.01 | 19.38 | 21.14 | 20.21 | 20.98 |
| 7:8 | 17.30 | 18.21 | 17.56 | 18.07 | 19.44 | 21.22 | 20.28 | 21.07 |
| 7:9 | 17.33 | 18.26 | 17.60 | 18.12 | 19.50 | 21.30 | 20.35 | 21.16 |
| 7:10 | 17.37 | 18.31 | 17.65 | 18.17 | 19.56 | 21.39 | 20.42 | 21.25 |
| 7:11 | 17.40 | 18.36 | 17.69 | 18.23 | 19.62 | 21.47 | 20.49 | 21.35 |
| 8:0 | 17.44 | 18.41 | 17.73 | 18.28 | 19.68 | 21.56 | 20.56 | 21.44 |
| 8:1 | 17.47 | 18.46 | 17.77 | 18.34 | 19.74 | 21.65 | 20.63 | 21.54 |
| 8:2 | 17.51 | 18.51 | 17.82 | 18.39 | 19.80 | 21.74 | 20.71 | 21.64 |
| 8:3 | 17.55 | 18.56 | 17.87 | 18.45 | 19.86 | 21.83 | 20.78 | 21.74 |
| 8:4 | 17.59 | 18.62 | 17.91 | 18.51 | 19.93 | 21.92 | 20.86 | 21.84 |
| 8:5 | 17.62 | 18.67 | 17.96 | 18.57 | 19.99 | 22.02 | 20.94 | 21.94 |
| 8:6 | 17.66 | 18.73 | 18.01 | 18.63 | 20.06 | 22.11 | 21.02 | 22.04 |
| 8:7 | 17.70 | 18.78 | 18.06 | 18.69 | 20.12 | 22.21 | 21.10 | 22.14 |
| 8:8 | 17.74 | 18.84 | 18.11 | 18.75 | 20.19 | 22.31 | 21.18 | 22.24 |
| 8:9 | 17.78 | 18.90 | 18.17 | 18.81 | 20.26 | 22.41 | 21.26 | 22.35 |
| 8:10 | 17.82 | 18.95 | 18.22 | 18.87 | 20.33 | 22.51 | 21.35 | 22.45 |
| 8:11 | 17.87 | 19.01 | 18.27 | 18.93 | 20.40 | 22.61 | 21.43 | 22.56 |
| 9:0 | 17.91 | 19.07 | 18.33 | 18.99 | 20.47 | 22.71 | 21.51 | 22.66 |
| 9:1 | 17.95 | 19.13 | 18.38 | 19.05 | 20.54 | 22.82 | 21.60 | 22.77 |
| 9:2 | 18.00 | 19.19 | 18.44 | 19.12 | 20.61 | 22.92 | 21.68 | 22.88 |
| 9:3 | 18.04 | 19.25 | 18.49 | 19.18 | 20.69 | 23.03 | 21.77 | 22.99 |
| 9:4 | 18.09 | 19.31 | 18.55 | 19.24 | 20.76 | 23.13 | 21.86 | 23.09 |
| 9:5 | 18.13 | 19.37 | 18.61 | 19.31 | 20.84 | 23.24 | 21.94 | 23.20 |
| 9:6 | 18.18 | 19.43 | 18.67 | 19.38 | 20.92 | 23.34 | 22.03 | 23.31 |
| 9:7 | 18.23 | 19.49 | 18.73 | 19.44 | 20.99 | 23.45 | 22.12 | 23.42 |
| 9:8 | 18.28 | 19.55 | 18.79 | 19.51 | 21.07 | 23.55 | 22.21 | 23.53 |
| 9:9 | 18.33 | 19.61 | 18.85 | 19.58 | 21.15 | 23.66 | 22.30 | 23.64 |
| 9:10 | 18.38 | 19.67 | 18.91 | 19.64 | 21.23 | 23.76 | 22.39 | 23.75 |
| 9:11 | 18.43 | 19.74 | 18.97 | 19.71 | 21.32 | 23.86 | 22.48 | 23.86 |

BMI, body mass index; IOTF, International Obesity Task Force; WHO, World Health Organization.

^a^WHO cut-off values for overweight are defined to pass through a BMI of 25 kg/m^2^ at the age of 19 years [17]. Overweight is defined as a BMI greater than the given value.

^b^IOTF cut-off values for overweight are defined to pass through a BMI of 25 kg/m^2^ at the age of 18 years [19]. Overweight is defined as a BMI equal to or greater than the given value.

^c^WHO cut-off values for obesity are defined to pass through a BMI of 30 kg/m^2^ at the age of 19 years [17]. Obesity is defined as a BMI greater than the given value.

^d^IOTF cut-off values for obesity are defined to pass through a BMI of 30 kg/m^2^ at the age of 18 years [19]. Obesity is defined as a BMI equal to or greater than the given value.
