# Supplementary material for: WHO European Childhood Obesity Surveillance Initiative: body mass index and level of overweight among 6–9-year-old children from school year 2007/2008 to school year 2009/2010
Source: BMC Public Health. 2014 Aug 7;14:806. doi: 10.1186/1471-2458-14-806 (PMC4289284; doi:10.1186/1471-2458-14-806)
Supplement: Supplementary file 6 — Additional file 6: Median and interquartile range (Q1–Q3) values of weight and BMI of boys and girls aged 6–9 years in COSI Round 2 (2009/2010), by age and country. (DOCX 62 KB) [file 12889_2014_6942_MOESM6_ESM.docx]

**Additional file 6** Median and interquartile range (Q1–Q3) values of weight and BMI of boys and girls aged 6–9 years in COSI Round 2 (2009/2010), by age and country

| Age group and country^‡^ | Weight* (kg) | | BMI^#^ (kg/m^2^) | |
| --- | --- | --- | --- | --- |
|  | Boys | Girls | Boys | Girls |
|  | Median (Q1–Q3) | | | |
| 6-year-olds |  |  |  |  |
| BEL | 22.5 (20.6–24.8) | 22.1 (20.1–24.6) | 15.5 (14.7–16.5) | 15.4 (14.5–16.6) |
| SVN | 23.9 (21.7–26.9) | 23.6 (21.2–26.5) | 15.5 (14.5–16.8) | 15.4 (14.4–16.9) |
| ESP | 23.9 (21.8–27.1) | 23.7 (21.5–26.8) | 16.4 (15.4–17.7) | 16.3 (15.1–18.1) |
| 7-year-olds |  |  |  |  |
| BEL | 24.9 (22.5–28.1) | 24.6 (22.1–28.1) | 15.8 (14.8–17.1) | 15.8 (14.7–17.4) |
| CZE | 25.0 (22.7–28.0) | 24.0 (21.9–28.0) | 15.9 (14.7–17.1) | 15.7 (14.6–17.2) |
| GRC | 28.7 (24.9–32.6) | 27.9 (24.6–32.8) | 17.1 (15.7–19.3) | 17.1 (15.7–19.4) |
| HUN | 25.4 (23.0–28.9) | 25.1 (22.3–29.4) | 15.7 (14.8–17.3) | 15.7 (14.6–17.8) |
| IRL | 25.4 (23.1–28.2) | 24.9 (22.3–27.9) | 16.0 (15.2–17.2) | 16.3 (15.1–17.8) |
| LVA | 26.4 (24.0–29.7) | 25.4 (22.7–28.8) | 16.0 (15.0–17.2) | 15.7 (14.6–17.1) |
| LTU | 26.4 (24.3–30.1) | 25.9 (23.3–29.6) | 15.9 (15.0–17.2) | 15.7 (14.6–17.2) |
| PRT | 25.8 (23.3–29.4) | 26.3 (23.1–30.0) | 16.2 (15.1–17.9) | 16.7 (15.3–18.5) |
| SVN | 26.7 (23.9–30.2) | 25.8 (23.2–29.7) | 16.0 (14.9–17.6) | 15.8 (14.6–17.5) |
| ESP | 27.4 (24.4–31.6) | 26.9 (23.5–31.1) | 17.0 (15.6–18.9) | 16.9 (15.4–19.0) |
| MKD | 26.0 (23.1–30.5) | 25.0 (22.2–29.5) | 16.2 (14.9–18.2) | 15.8 (14.6–17.7) |
| 8-year-olds |  |  |  |  |
| BEL | 28.2 (25.6–31.7) | 28.0 (25.1–31.9) | 16.0 (15.1–17.4) | 16.1 (15.0–17.8) |
| ITA | 30.8 (26.9–36.3) | 30.2 (26.2–35.6) | 17.3 (15.8–19.9) | 17.3 (15.6–19.8) |
| NOR | 29.3 (26.2–33.1) | 28.8 (25.7–32.5) | 16.4 (15.3–17.9) | 16.5 (15.3–18.1) |
| SVN | 30.1 (26.7–35.0) | 29.6 (26.2–34.7) | 16.7 (15.4–18.9) | 16.6 (15.2–18.8) |
| ESP | 30.6 (27.0–35.7) | 30.1 (26.7–35.1) | 17.3 (15.9–19.6) | 17.4 (15.8–19.6) |
| 9-year-olds |  |  |  |  |
| BEL | 31.2 (27.9–35.9) | 31.2 (27.6–36.6) | 16.5 (15.3–18.4) | 16.6 (15.3–18.8) |
| GRC | 36.5 (31.9–43.9) | 36.2 (30.8–43.0) | 18.8 (16.9–21.8) | 18.7 (16.4–21.5) |
| IRL | 31.9 (28.3–36.2) | 31.0 (27.1–36.0) | 17.2 (15.9–18.7) | 17.1 (15.5–19.0) |
| ITA | 32.2 (28.1–38.2) | 31.5 (27.3–37.2) | 17.5 (15.9–20.2) | 17.3 (15.6–19.8) |
| LTU | 33.1 (29.7–37.8) | 31.9 (28.8–37.0) | 16.7 (15.5–18.5) | 16.4 (15.2–18.2) |
| SVN | 32.5 (28.6–37.6) | 31.8 (28.2–37.8) | 17.0 (15.7–19.2) | 16.9 (15.6–19.6) |
| ESP | 34.5 (30.5–40.3) | 33.8 (29.6–39.8) | 18.1 (16.4–20.5) | 18.1 (16.2–20.5) |

Abbreviations: BMI, body mass index; BMI/A, BMI-for-age; COSI, Childhood Obesity Surveillance Initiative; Q1, first quartile; Q3, third quartile; W/A, weight-for-age.

^‡^The country codes refer to the International Organization for Standardization (ISO) 3166-1 Alpha-3 country codes and countries were listed in alphabetical order by their full names: BEL, Belgium (Flanders); CZE, Czech Republic; GRC, Greece; HUN, Hungary; IRL, Ireland; ITA, Italy; LVA, Latvia; LTU, Lithuania; NOR, Norway; PRT, Portugal (all regions except Madeira); SVN, Slovenia; ESP, Spain; MKD, the former Yugoslav Republic of Macedonia.

*Body weight was adjusted for clothes worn when measured and children with a W/A Z-score <–6 or >+5 were excluded.

^#^Body weight was adjusted for clothes worn when measured and children with a BMI/A Z-score <–5 or >+5 were excluded.
